# AMPK-p38 axis converts human pluripotent stem cells to naive state

**DOI:** 10.1016/j.isci.2026.115569

**Published:** 2026-04-01

**Authors:** Zhennan Yang, Yajing Liu, Huaigeng Xu, Junko Yamane, Akitsu Hotta, Wataru Fujibuchi, Jun K. Yamashita

**Affiliations:** 1 Department of Cellular and Tissue Communications, Graduate School of Medicine, the University of Tokyo, Tokyo 113-8655, Japan; 2 The Department of Cell Growth and Differentiation, Center for iPS Cell Research and Application (CiRA), Kyoto University, Kyoto 606-8507, Japan; 3 The Department of Life Science Frontiers, Center for iPS Cell Research and Application (CiRA), Kyoto University, Kyoto 606-8507, Japan

**Keywords:** cell biology, stem cells research, developmental biology

## Abstract

Pluripotent stem cells (PSCs) exist in either a “primed” state or a “naive” state. While several protocols are available to convert primed human PSCs (hPSCs) to the naive-state hPSCs, they often require multiple exogenous factors. Here, we show that the activation of AMP-activated protein kinase (AMPK) or its downstream p38 alone induces naive conversion. Primed hPSCs cultured with activated AMPK-p38 displayed key naive features, including naive marker expression, three-germ-layer differentiation, epigenomic resetting, and increased mitochondrial activity. An AMPK activator—5-aminoimidazole-4-carboxamide-1-β-D-ribofuranoside (AICAR)—synergistically enhances the naive conversion efficiency of reported conversion protocols. Single-cell RNA sequencing (scRNA-seq) with RNA velocity analyses and Totem trajectory mapping identified an intermediate state bridging the primed and naive states. These cells showed three upregulated gene groups: (1) pluripotency genes (e.g., *Pou5f1* and *Nanog*), (2) naive state-related genes (e.g., *Dnmlt3l* and *Alpg*), and (3) differentiation-suppressive genes (e.g., *Rest* and *Hhla1*). These findings establish a simple induction method that illuminates underlying mechanisms and enables broad applications through efficient naive conversion.

## Highlights

• AMPK activation by AICAR or metformin converts primed hPSCs to naive pluripotent state
• p38 MAPK is a critical downstream mediator of AMPK-induced naive conversion
• AICAR synergistically enhances naive conversion efficiency of existing protocols
• scRNA-seq reveals an intermediate state bridging primed and naive pluripotency

## Graphical abstract

**Figure:**
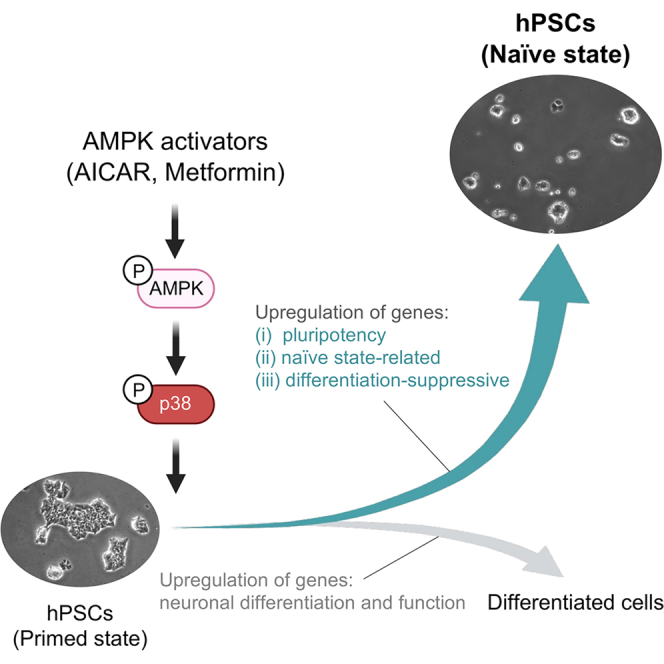

## Introduction

Pluripotent stem cells (PSCs) are generally classified into two developmental states: “primed” and “naive.” Typically, human PSCs (hPSCs) are in the primed state, whereas mouse embryonic stem cells (ESCs) predominantly remain in the naive state.^1^^,^^2^^,^^3^ The naive state closely resembles the inner-cell mass (ICM) of pre-implantation blastocysts, whereas the primed state is more akin to the post-implantation epiblast.^3^^,^^4^^,^^5^^,^^6^^,^^7^ The primed-state cells are considered incapable of chimera formation with blastocysts, while the naive-state cells are considered to have this capability.^8^^,^^9^ In addition, naive-state PSCs express pluripotent genes, including naive state-specific markers, and exhibit one of the lowest levels of DNA methylation during the whole developmental process.^10^^,^^11^^,^^12^^,^^13^ Recent studies have demonstrated that transient naive reprogramming to the lowest methylation stage can correct functional and epigenetic anomalies in human induced pluripotent stem cells (hiPSCs), normalizing DNA methylation and improving differentiation potential.^14^ Thus, research on naive-state PSCs and the mechanism of their conversion leads to better maintenance of pluripotency and the ability to contribute to chimera formation, which is critical for regenerative medicine and developmental biology research. Several groups have reported protocols for converting hPSCs from the primed state to naive state. These studies have used various combinations of small molecules and cytokines (Table S1)^9^^,^^15^^,^^16^^,^^17^^,^^18^^,^^19^^,^^20^^,^^21^^,^^22^ or an HDAC inhibitor, which broadly alters cellular epigenetic status^19^ to mediate the conversion process. Despite these advances, the identity of a singular key regulatory axis that drives naive conversion in hPSCs remains elusive. Recently, our group demonstrated that the activation of 5′-adenosine monophosphate-activated protein kinase (AMPK) with single small-molecule activators can convert primed mouse epiblast stem cells (EpiSCs) into naive mouse ESCs. Treatment of mouse EpiSCs with AMPK activators such as 5-aminoimidazole-4-carboxamide-1-β-D-ribofuranoside (AICAR), A769662, or metformin resulted in the upregulation of pluripotency and naive-specific genes, ultimately producing naive cells with germline transmission potential.^23^ AMPK is activated under energy stress conditions such as caloric restriction and plays a fundamental role in metabolic homeostasis. Here, we report the effects of AMPK and its downstream effector p38 on naive conversion of hPSCs. Adding AICAR or metformin to the primed hPSCs cultured in various naive maintenance media^22^^,^^24^ induced naive-like cells. Notably, AICAR markedly boosts the conversion efficiency observed in FXGAY/A condition, an updated 5i/L/A protocol^22^ (see STAR Methods). We further demonstrate that p38 activation alone is sufficient to trigger naive conversion in hPSCs. Single-cell RNA sequencing (scRNA-seq) together with trajectory analyses of AMPK-p38-mediated naive conversion process revealed an intermediate state whose transcriptome suggests that genes previously implicated in naive conversion (e.g., in mouse studies) alone are insufficient to confer naive identity. Instead, successful conversion requires coordinated upregulation of three gene groups: (1) pluripotency genes, (2) naive state-related genes, and (3) differentiation-suppressive genes. Thus, our findings identify the AMPK-p38 axis as a singular signaling pathway capable of inducing a naive state in hPSCs, which can potently enhance other previously reported naive-conversion methods. This simple and efficient naive conversion method will be valuable for dissecting the regulatory networks that underpin naive pluripotency and could considerably advance applications of naive-state cells to both chimera-based developmental studies and the generation of high-quality PSCs for regenerative medicine.

## Results

### AICAR converts hPSCs to the naive state

**Figure 1:**
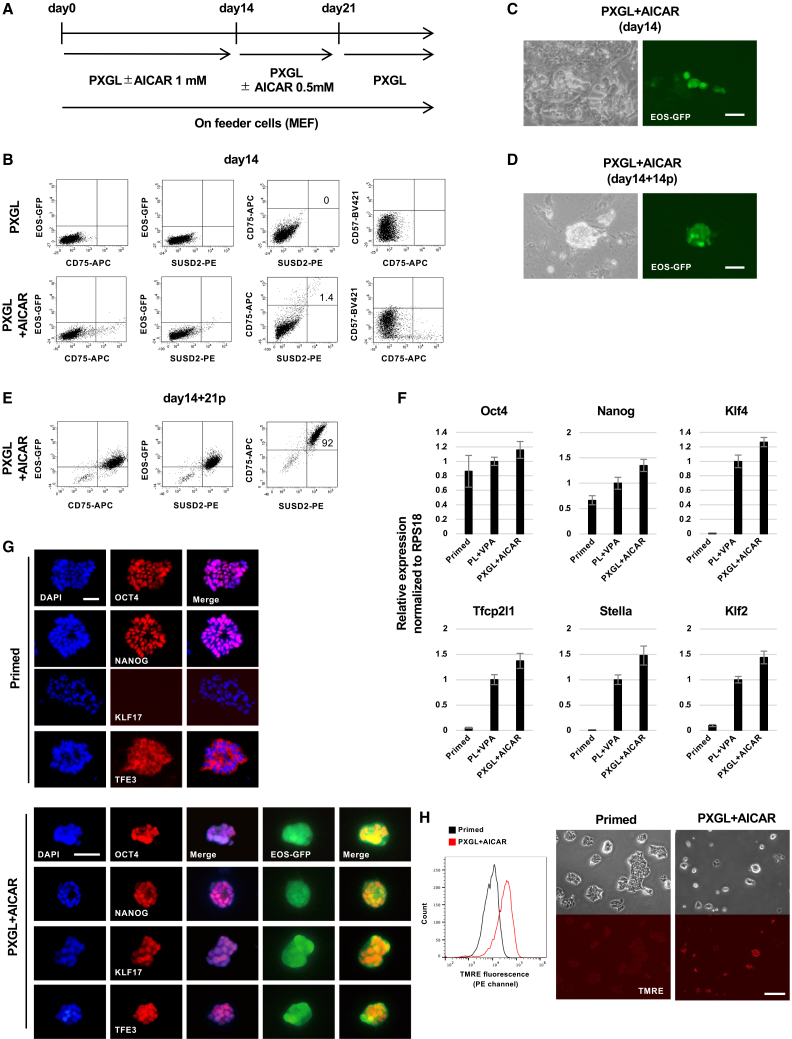
Naive conversion with AICAR (A) Naive conversion protocol using AICAR. PXGL; PD0325901 (1 μM), XAV939 (2 μM), Go6983 (2 μM), and human LIF (10 ng/mL) in Ndiff227 medium. (B) Flow cytometry analysis of EOS-GFP, SUSD2, CD75, and CD57 expression (day 14). (C) Appearance of EOS-GFP-positive cell clusters induced by AICAR (day 14). (D) EOS-GFP-positive naive-like colony after expansion in PXGL (day 14 + 14p). (E) Flow cytometry analysis of EOS-GFP, SUSD2, and CD75 expression (day 14 + 21p). (F) RT-qPCR analysis of sorted SUSD2^+^CD75^+^ cells and parental primed human ESCs (H9-EOS). AICAR, day 14 + 13p; VPA, day 9 + 4p. Error bars: SD of technical triplicates. (G) Immunostaining for OCT4, NANOG, KLF17, and TFE3 of primed and AICAR-induced cells. AICAR, day 14 + 14p. (H) AICAR-treated cells showed increased TMRE fluorescence, as detected by both flow cytometry (PE channel) and fluorescence microscopy. Scale bars: 100 μm in (C, D, and H) and 50 μm (G). AICAR, day 14 + 16p. See also Figures S1 and S2 and Table S1.

We previously reported that the treatment with AMPK activators such as AICAR or metformin or the expression of a constitutively active form of p38 (CA-p38) can maintain the naive state of mouse ESCs and convert mouse EpiSCs to naive mouse ESCs.^23^^,^^25^ On the basis of these findings, we investigated whether the AMPK activators AICAR, metformin, or CA-p38 could convert primed hPSCs to their naive state. As an initial attempt, we treated human ESCs and human iPSCs^26^^,^^27^ with AICAR. While naive mouse ESCs can be fully maintained *in vitro* by culturing in a medium containing two small molecule inhibitors (2i) of kinases (MEK and GSK3) and the leukemia inhibitory factor (LIF) 2iL,^28^^,^^29^^,^^30^ naive hPSCs generally require mouse embryonic fibroblast (MEF) feeder cells and additional modifications such as the Wnt inhibitor XAV939.^24^ In this study, we mainly employed PXGL medium, consisting of PD0325901 (1 μM), XAV939 (2 μM), Go6983 (2 μM), and human LIF (10 ng/mL) in Ndiff227 medium^24^ with MEF feeder cells, for the naive conversion of human cells (Figure 1A). We first confirmed that when primed hPSCs were cultured with PXGL medium (with MEFs) alone, naive cells did not emerge (Figure 1B). We then evaluated the effect of AMPK activation in PXGL (with MEFs) culture condition. The emergence of naive hPSCs was initially monitored by EOS-GFP reporter expression as a naive-state marker.^31^^,^^32^

We generated hPSCs expressing the EOS-GFP reporter gene (see STAR Methods), using H9 human ESCs (H9-EOS) and Ff-I14 human iPSCs (Ff-I14-EOS). When H9-EOS cells were treated with valproic acid (VPA), which has been reported to induce a primed-to-naive state transition in hPSCs,^19^ GFP-positive cells were successfully induced (Figure S1A), indicating the validity of the EOS-expressing cell system. Upon treatment with 1 mM AICAR in the PXGL medium for 14 days, the emergence of small GFP-positive cell clusters was observed (Figure 1C). Flow cytometry analysis confirmed that only a small but distinct population of GFP-positive cells appeared in the AICAR-treated cells compared to those treated with PXGL medium alone (Figure 1B). Most of the cells were negative for CD57, a marker of primed state, while a few cells began to be positive (less than 2%) for the naive-state markers CD75 and SUSD2.^24^^,^^33^ Following the emergence of GFP-positive cells (14 days of induction), the cells were passaged and cultured for an additional 1 week with a reduced AICAR concentration (0.5 mM) (Figure 1A). We then removed AICAR and continued to expand the cells in the naive maintenance PXGL medium alone. GFP-positive cells could propagate for over 4 months in the PXGL condition without AICAR, forming naive-like dome-shaped colonies (Figure 1D). Their doubling time was 4–5 days (Figure S1B). The CD75^+^/SUSD2^+^/GFP^+^ cell population was highly enriched after passages in the PXGL condition (Figure 1E). Quantitative reverse-transcription PCR (RT-qPCR) analysis of fluorescence-activated cell sorting (FACS)-purified CD75^+^/SUSD2^+^ naive-like cells showed that the pluripotency markers Oct4 and Nanog were expressed in both primed H9-EOS and VPA- or AICAR-induced naive-like cells. In contrast, the naive state markers, Klf4, Tfcp2l1, Stella, and Klf2 expressed only in the naive-like cells (VPA- or AICAR-induced cells) but not in primed cells (Figure 1F). Immunofluorescence staining also revealed the expression of OCT4 and NANOG in both primed-state H9-EOS cells and the naive-like cells, while the naive state marker KLF17 expressed only in the naive-like cells. Nuclear translocation of TFE3 has been documented in naive-state cells.^34^ The nuclear TFE3 localization was observed in VPA- or AICAR-induced cells, as opposed to cytoplasmic localization in primed H9-EOS cells (Figure 1G). An increase in mitochondrial activity, a feature commonly associated with naive PSCs, was observed in AICAR-induced cells in comparison to primed H9-EOS cells, as indicated by tetramethyl-rhodamine methyl ester (TMRE) staining^31^^,^^35^ (Figure 1H). These findings suggest that AICAR-induced cells exhibit various hallmarks of the naive state. Furthermore, hiPSCs (Ff-I14-EOS) also successfully gave rise to CD75^+^/SUSD2^+^/GFP^+^ dome-shaped naive cell-like colonies by AICAR treatment (Figure S1C). In addition, we confirmed that the 1231A3 hiPSC line also responded to AICAR treatment, with the appearance of CD75^+^/SUSD2^+^ populations, which further supported the reproducibility of our approach across multiple human PSC lines (Figure S1C).

Recently, several novel conditions for naive maintenance and induction have been reported, including AXGY and FXGY for maintenance and FXGAY/A for induction.^22^ AXGY conditions consist of the pan-RAF inhibitors AZ628 (5 μM), XAV939 (2 μM), Gö6983 (2 μM), and Y-27632 (10 μM), while FXGY conditions include the FGFR1 inhibitors PD166866 (1 μM), XAV939 (2 μM), Gö6983 (2 μM), and Y-27632 (10 μM). We confirmed that AXGY and AXG alone could not induce naive conversion, but the addition of both AICAR and metformin induced a CD75^+^/SUSD2^+^ naive population in both conditions (Figures S1D–S1F), suggesting that AMPK activators work in a variety of culture conditions. FXGAY/A condition, a previously developed naive induction method, consists of the FGFR1 inhibitors PD166866 (1 μM), XAV939 (2 μM), Gö6983 (2 μM), AZ628 (5 μM), Y-27632 (10 μM), and Activin A (10 ng/mL). FXGAY/A represents an improved method over the 5i/L/A protocol, as it omits the MEK1/2 inhibitor PD0325901, which has been associated with genomic instability and loss of imprinting.^2^^,^^13^^,^^36^^,^^37^ Then, we tested the combinatory effect of AICAR with FXGAY/A (Figure S2A). FXGA, which lacks Y-27632 and Activin A in FXGAY/A, did not induce naive conversion. Addition of AICAR or Activin A to FXGA showed comparable appearance of CD75^+^/SUSD2^+^ populations. Simultaneous addition of AICAR and FXGAY/A synergistically enhanced the naive conversion efficiency, increasing CD75^+^/SUSD2^+^ cells on day 7 by 4.1-fold (mean). These results indicated that AMPK promotes naive conversion through a pathway distinct from Activin A and, when activated, synergistically boosts the efficiency of conventional protocols.

### Differentiation ability of AICAR-induced naive hPSCs

**Figure 2:**
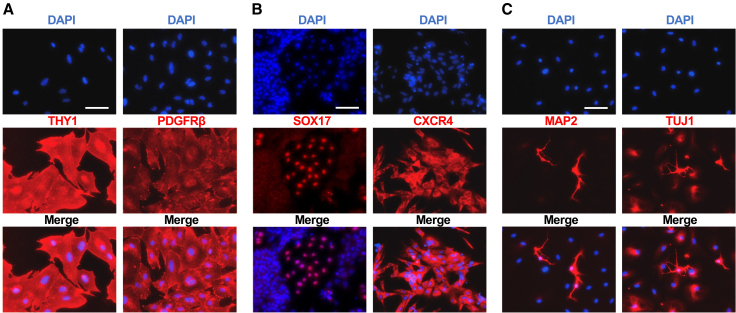
Differentiation of AICAR-induced naïve-like cells (A) Representative immunofluorescence staining images for the mesoderm markers THY1 and PDGFR-β. (B) Representative immunofluorescence staining images for the endoderm markers SOX17 and CXCR4. (C) Representative immunofluorescence staining images for the ectoderm markers MAP2 and TUJ1. Scale bars: 100 μm. See also Figure S5.

We next assessed the functional properties of the AICAR-induced naive-like cells. As a first step, we evaluated their *in vitro* differentiation potential. Because the differentiation protocols we used were originally designed for primed PSCs, the AICAR-induced naive-like cells were “re-primed” when cultured in AK02N (a primed cell medium) for more than three passages. We then examined their capacity to differentiate into the three germ layers. For mesoderm differentiation, Thy-1 cell surface antigen (THY1^+^) and platelet-derived growth factor receptor beta (PDGFR-β^+^) cells were efficiently obtained (all 205 of 205 and 99 of 99 DAPI-positive nuclei, respectively), using a modified DD protocol^38^ (Figure 2A). Endoderm cell appearance induced by the treatment with Activin A and Wnt3A^39^ was confirmed with Sox17-positive (106 of 2109 DAPI-positive nuclei) and CXCR4-positive (129 of 133 nuclei) cells (Figure 2B). Neural differentiation achieved using Noggin and SB431542^40^ was demonstrated with TUJ1-positive (all 174 DAPI-positive nuclei) and MAP2-positive (14 of 177 nuclei) cell appearance (Figure 2C).

### Epigenomic status of AICAR-induced naive hPSCs

**Figure 3:**
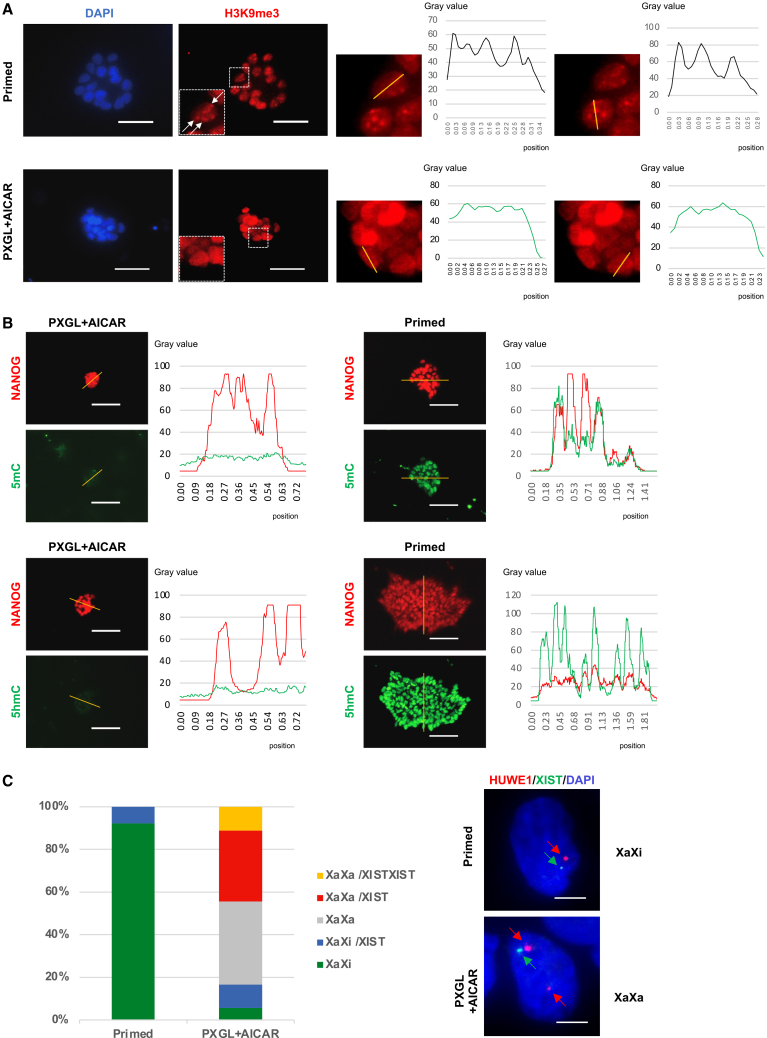
Epigenomic status of AICAR-induced naive hPSCs (A) Immunostaining of H3K9me3 with intensity and nuclear distribution profiles analyzed using ImageJ. (B) Immunostaining for 5mC, 5hmC, and NANOG. Quantification of global 5mC and 5hmC fluorescence intensity, performed using ImageJ. (C) X chromosome status of parental primed and AICAR-induced cells analyzed by RNA FISH for HUWE1 and XIST. H9-EOS hPSCs treated with AICAR (day 14 + 14p) are shown in (A) and (B), whereas 1231A3 hPSCs treated with AICAR (day 14 + 10p) are shown in (C). Scale bars: 50 μm (A), 100 μm (B), and 5 μm (C). See also Figure S4.

To further verify that the AICAR-induced cells exhibited defining features of the naive state, we examined their epigenomic characteristics in comparison with primed cells.^37^ Histone 3 lysine 9 trimethylation (H3K9me3) is a hallmark of heterochromatin, and the formation of distinct H3K9me3 foci is a characteristic feature of primed-state PSCs but not of the naive state.^31^ Consistent with this, H3K9me3 foci were observed in primed H9-EOS cells but not in AICAR-induced naive-like cells (Figure 3A). Human ICM cells, naive mouse ES cells, and naive hPSCs are known to exhibit global DNA hypomethylation, whereas primed mouse EpiSCs and primed hPSCs generally show higher DNA methylation levels.^12^^,^^31^^,^^41^^,^^42^

Indeed, immunofluorescence for 5-methylcytosine (5mC) and 5-hydroxymethylcytosine (5hmC) revealed reduced signals in AICAR-induced naive-like cells compared with primed controls (Figure 3B).

Additionally, naive hPSCs often maintain two active X chromosomes (XaXa), whereas primed cells typically carry one inactive X chromosome (XaXi).^19^^,^^43^ To investigate this in our female cell line, we performed RNA fluorescence *in situ* hybridization (RNA FISH) to detect *HUWE1*, a gene expressed from the active X chromosome, and *XIST*, a non-coding RNA transcribed from the inactive X chromosome (Figure 3C). While primed cells predominantly displayed a monoallelic HUWE1 pattern (consistent with XaXi) accompanied by either no XIST (XaXi noXIST) or a single XIST signal (XaXi monoXIST), AICAR-induced naive cells contained a subpopulation exhibiting biallelic HUWE1 (indicating two active X chromosomes). These cells also showed absent, monoallelic, or biallelic XIST, consistent with previously reported naive-state features.

Taken together, these results demonstrated that simply adding AICAR to naive maintenance conditions is sufficient to convert primed hPSCs into naive-like cells exhibiting distinct naive criteria, including hallmarks of mitochondrial activity, differentiation potential, and characteristic epigenomic status.

### p38 acts downstream of AICAR and can convert hPSCs to naive state

**Figure 4:**
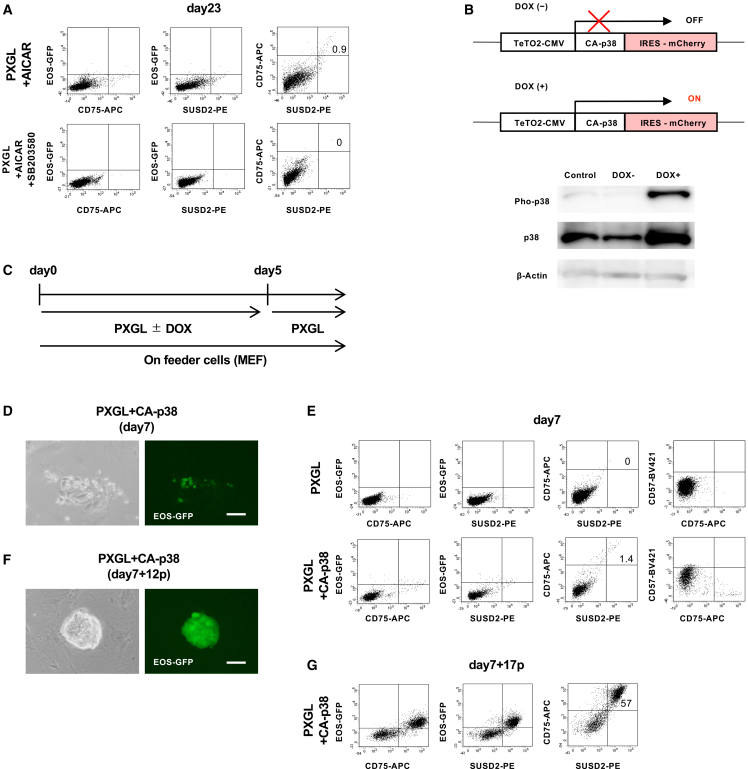
Involvement of p38 in AMPK-mediated naive conversion (A) Flow cytometry analysis of EOS-GFP, SUSD2, and CD75 expression under treatment with a p38 inhibitor, SB203580 (10 μM) (day 23). (B) Tet-inducible CA-p38 expression system (top). Western blotting for p38 proteins after Dox treatment (bottom); β-actin serves as the loading control . (C) Naive conversion protocol with p38 activation. (D) Appearance of EOS-GFP-positive cell cluster induced by CA-p38 (day 7). (E) Flow cytometry analysis of EOS-GFP, SUSD2, CD75, and CD57 expression after induction of CA-p38 (day 7). (F) EOS-GFP-positive naive-like colony after expansion in PXGL (day 7 + 12p). (G) Flow cytometry analysis of EOS-GFP, SUSD2, and CD75 expression after expansion in PXGL (day 7 + 17p). Scale bars: 100 μm. See also Figures S2 and S3.

Given that AMPK regulates several downstream pathways influencing metabolism, cell growth, autophagy, and other biological processes,^44^^,^^45^^,^^46^ we previously reported that p38 is a critical downstream target of AMPK for maintaining and inducing the naive state in mouse PSCs.^23^^,^^25^ To determine whether p38 plays a similar role in human cells, we examined its function in AICAR-driven naive conversion of HPSCs. First, we found that treating hPSCs with AICAR in combination with a p38 inhibitor, SB203580, blocked the AICAR-driven naive induction (Figure 4A), indicating that p38 activity is required for this process. Next, to confirm the capacity of p38 to induce naive conversion, we generated hPSCs harboring tetracycline-inducible (Tet-ON) CA-p38 in H9-EOS cells (CA-p38-H9-EOS).^25^^,^^47^ Upon doxycycline (Dox) treatment, p38 became phosphorylated and activated (Figures 4B and S3A). After 5 days of Dox treatment in PXGL medium (Figure 4C), clusters of EOS-GFP-positive cells emerged (Figure 4D). Flow cytometry detected a small but distinct population (<2%) of CD75^+^/SUSD2^+^/CD57^-^/GFP^+^ cells (Figure 4E). We then cultured these CA-p38-induced GFP^+^ cells in PXGL medium on MEF feeders without Dox, where they formed dome-shaped colonies (Figure 4F). We subsequently transferred them to feeder-free PXGL, maintaining a stable doubling time of approximately 4 days for over 4 months (Figure S3B). By passage 17 (day 7 + 17p), flow cytometry confirmed robust EOS-GFP, CD75, and SUSD2 expression (Figure 4G). FACS-sorted CD75^+^/SUSD2^+^ cells showed Oct4 and Nanog levels comparable to those of the parental primed line (H9-EOS-GFP) but markedly higher expression of the naive markers Klf4, Tfcp2l1, Stella, and Klf2 (Figure S3C).

Additionally, CA-p38-induced cells showed protein-level expression of pluripotency and naive markers, along with the nuclear localization of TFE3 (Figure S3D). They also displayed enhanced mitochondrial activity, as evidenced by TMRE staining (Figure S3E) and epigenomic features consistent with a naive state (Figure S4). After re-priming, these CA-p38-induced cells retained the capacity to differentiate into the three embryonic germ layers (Figure S5). For ectoderm differentiation, MAP2-positive (31 of 157 DAPI-positive nuclei) and TUJ1-positive (99 of 498 DAPI-positive nuclei) cells were obtained. For endoderm differentiation, CXCR4-positive (163 of 164 DAPI-positive nuclei) and SOX17-positive (110 of 117 DAPI-positive nuclei) cells were detected. For mesoderm differentiation, PDGFR-β^+^ (103 of 139 DAPI-positive nuclei) and THY1^+^ (101 of 101 DAPI-positive nuclei) cells were observed. Together, these findings demonstrated that activating p38 alone is sufficient to convert human ESCs to a naive state in the PXGL condition. We next examined whether p38 signaling is also required for AICAR-mediated naive conversion under other culture conditions. When hPSCs were induced with AICAR in the AXG medium, the converted population was not stably maintained; therefore, the culture was switched to FXGL, which better supported the expansion of CD75^+^/SUSD2^+^ naive-like cells (Figure S2B). Under this condition, co-treatment with the p38 inhibitor SB203580 markedly reduced the appearance of CD75^+^/SUSD2^+^ cells, indicating that p38 activity is required for AICAR-driven naive conversion beyond the PXGL condition. In addition, inhibition of the p38 pathway significantly decreased the naive conversion efficiency in the FXGAY/A condition, a previously reported Activin A-dependent induction system (mean, 0.5-fold; *p* < 0.05; Figure S2A). Together, these results demonstrated that p38 serves as a common downstream mediator essential for naive conversion across distinct induction protocols, including both AICAR- and Activin A-dependent pathways.

### Global gene expression analysis of primed- and naive-state hPSCs

**Figure 5:**
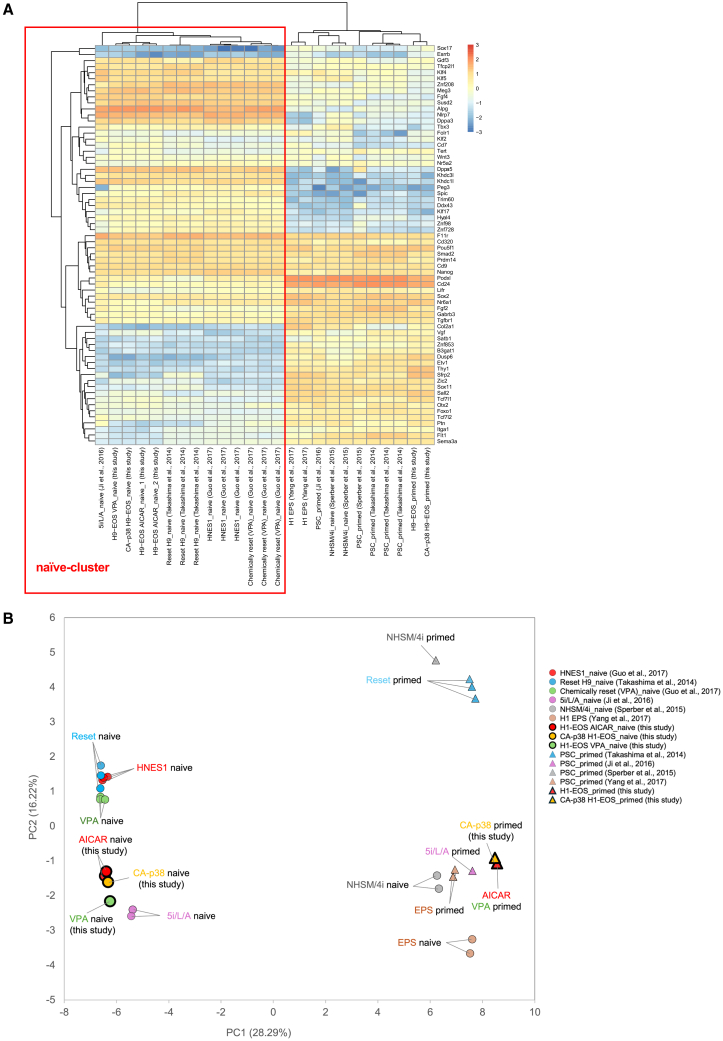
Comparative expression analysis of primed- and naive-state hPSCs (A) Heatmap of RNA expression for a selected set of naive- and primed-associated genes. The red box highlights the cluster of naive-like cells. VPA, day 9 + 12p; CA-p38, day 7 + 12p; AICAR, day 14 + 14p and day 14 + 15p. (B) PCA of the gene expression profiles using the same gene set as in (A), illustrating the relative distribution of primed and naive cell populations.

To assess the global transcriptomic similarity of our induced naive-state PSCs to previously reported naive and ICM-derived profiles, we performed bulk RNA-seq. Transcriptomic data from AICAR-, CA-p38-, and VPA-induced naive cells, together with their respective parental primed cells, were compared with published datasets of human naive ES cells (HNES1)^48^ derived from the human ICM, as well as with datasets of naive hPSCs converted from primed cells by using various protocols^17^^,^^19^^,^^31^^,^^49^^,^^50^ (see STAR Methods). A heatmap of 66 genes associated with naive and primed pluripotency showed that AICAR- and CA-p38-induced naive cells shared transcriptional profiles similar to those of previously reported naive cells, including HNES1 (Figure 5A). In particular, our AICAR- and CA-p38-induced naive hPSCs, together with VPA-induced naive cells,^19^ Reset cells,^31^ and 5i/L/A-induced naive cells,^17^ formed a cluster distinct from their respective parental primed cells. Consistently, principal-component analysis (PCA) of the same gene set (Figure 5B) showed that the Reset and VPA-induced naive cells clustered closely with HNES1. Notably, AICAR- and CA-p38-induced naive cells in our study exhibited an expression profile even closer to HNES1 than the VPA-induced naive cells generated in-house. Collectively, these findings demonstrated that our AICAR- and CA-p38-induced naive hPSCs reach a transcriptional state highly comparable to that of previously reported naive cells, including those derived from the human ICM.

### scRNA-seq analysis for naive conversion process

**Figure 6:**
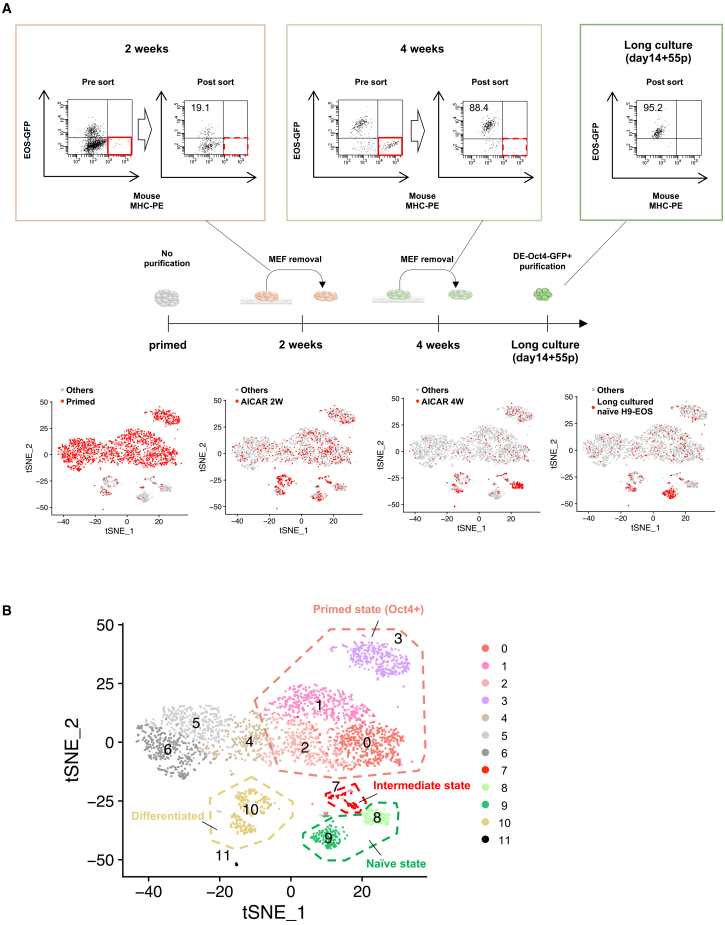
Single-cell RNA-seq analysis for naïve conversion process (A) t-SNE plot of all cell populations during naive conversion. Cells are colored according to sample origin: primed H9-EOS, AICAR (2 weeks), AICAR (4 weeks), and long-term cultured naive H9-EOS. (B) Twelve clusters (clusters 0–11) were identified from the top 37,273 most highly variable genes, using PCA dimension = 37, k = 50, and resolution = 2 in the Seurat package (v.4.0.1) for R (v.4.0.3). See also Figures S6 and S7.

**Figure 7:**
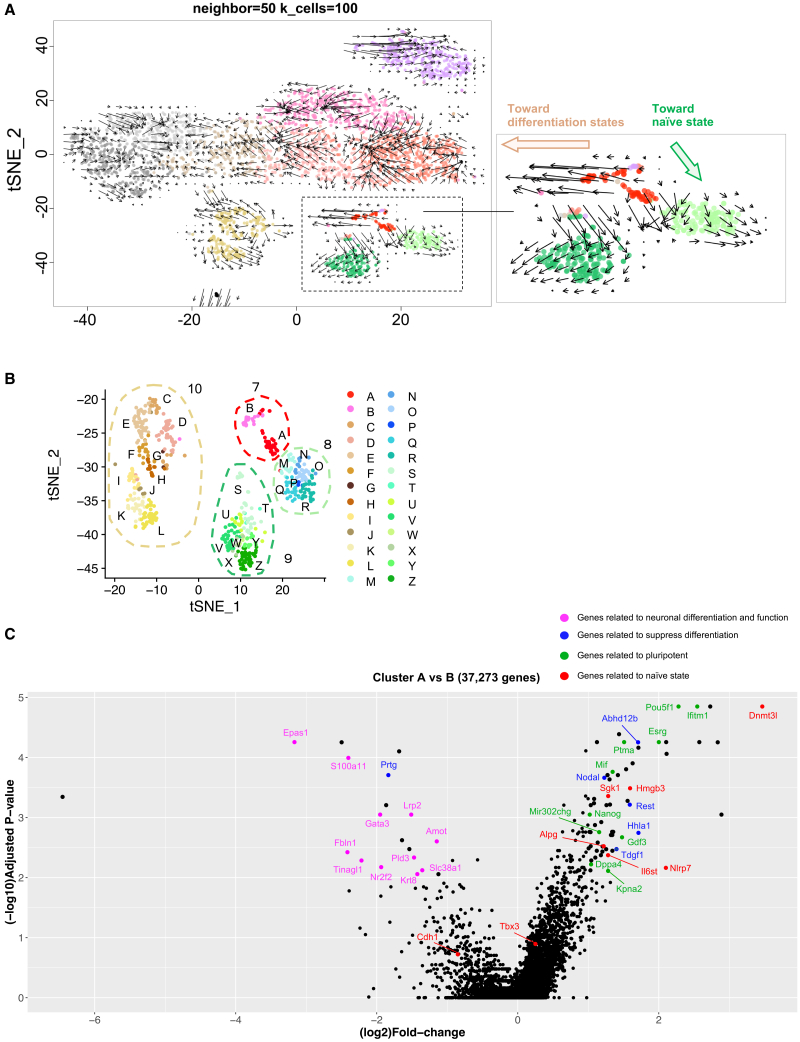
Transcriptional dynamics during naive conversion (A) RNA velocity analysis of all samples, where each vector’s direction indicates the predicted transcriptional trajectory. (B) Twenty-six clusters (clusters A–Z) were identified from the same dataset, using PCA dimension = 37, k = 50, and resolution = 5 in Seurat. (C) Volcano plot comparing gene expression between cluster A and cluster B. The *x* axis (log_2_ scale) represents fold change, and the *y* axis (log_10_ scale) shows the adjusted *p* value. Genes associated with the naive state are in red, pluripotency-related genes are in green, differentiation suppression-related genes are in blue, and neuronal differentiation/function-associated genes are in purple. See also Figures S7 and S8.

**Figure 8:**
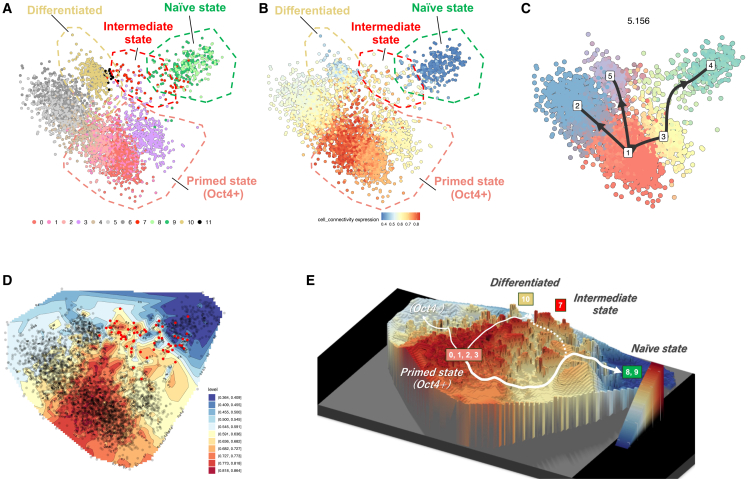
Trajectory analysis of naive conversion using Totem framework (A) Two-dimensional MDS colored by the 12 original t-SNE clusters, placing cluster 7 between primed, naive, and ectoderm-like groups. (B) Cell-connectivity map of the MDS projection; warmer tones indicate higher transition potential, highest around cluster 7. (C) A selected clustering among the top 20 results based on the VRC score in Totem. Arrows indicate potential trajectories when cluster 1 is set as the initial cluster. (D) Contour plot of interpolated cell connectivity values. Original cell positions are indicated by black circles, with cells from cluster 7 highlighted in red circles. (E) Three-dimensional connectivity landscape. Solid white line traces the main primed→naive route; dashed line shows an alternative path through the high-connectivity region (chiefly cluster 7), linking the naive (8 and 9) and ectoderm-like (10) states.

To gain mechanistic insight into the progression of naive conversion, we conducted scRNA-seq to trace transcriptional trajectories from the primed to the naive state. We analyzed four cell populations: primed H9-EOS grown in StemFit AK02N feeder-free culture (“primed”), AICAR+PXGL-treated cells at 2 weeks (AICAR 2 weeks) and 4 weeks (AICAR 4 weeks), and long-term feeder-free culture (over 10 passages) of AICAR-induced naive hPSCs (“long culture”). In total, 3,314 cells were analyzed (Figure 6A). The AICAR 2-week and 4-week samples were initially grown on MEF feeders; so, we depleted mouse MHC^+^ MEFs via FACS to isolate human (mouse MHC^−^) cells. For the long-cultured naive H9-EOS, we sorted EOS-GFP^+^ cells to achieve >95% purity. We then applied t-distributed stochastic neighbor embedding (t-SNE) clustering to group these cells into 12 populations (clusters 0–11; Figure 6B). Clusters 0–3 were enriched in primed H9-EOS cells expressing pluripotency markers such as Pou5f1 and Nanog (Figure S6A) and were, thus, classified as primed PSC populations. The AICAR 4-week sample (88.4% EOS-GFP^+^ after MEF depletion) predominantly occupied cluster 8, whereas the long-cultured naive H9-EOS populated cluster 9. Both clusters expressed pluripotency genes (e.g., *Pou5f1* and *Nanog*)^51^^,^^52^ and naive markers (e.g., Susd2 and Dppa3)^24^^,^^53^ (Figure S6B), identifying them as naive-state PSCs. By contrast, the AICAR 2-week sample showed only 19.1% EOS-GFP^+^ cells (partial or incomplete naive conversion) and primarily segregated into clusters 7 and 10. Cluster 10 expressed neural markers (Map2 and Pax6)^54^^,^^55^ but a few endoderm or mesoderm markers, suggesting an ectoderm-like lineage (Figure S6C). We suspect that Ndiff227 medium, known to favor neural differentiation, contributed to the ectodermal identity of cluster 10. Meanwhile, cluster 7 appeared as an intermediate state bridging the primed (clusters 0, 1, 2, and 3) and naive (clusters 8 and 9) states, supported by the presence of critical “naive inducer” genes such as *Cdh1* and *Tbx3*^56^^,^^57^ but a lack of several naive-specific genes (e.g., *Dppa5* and *Klf4*; Figures S6B, S6E, and S7A). Notably, RNA velocity analysis revealed a bifurcation in cluster 7 into two subpopulations: one moving toward naive clusters (8 and 9) and the other toward neural fates (clusters 10 and 11) (Figure 7A). We next explored gene expression dynamics in cluster 7 through a second t-SNE restricted to clusters 7–11, subdividing cluster 7 into subclusters A and B (Figure 7B). Volcano plots indicated that subcluster B, which showed a tendency toward neural lineages, upregulated neural differentiation genes (e.g., *Epas1* and *Gata3*).^58^^,^^59^ In contrast, subcluster A shifted toward the naive fate, expressing pluripotency genes (e.g., *Pou5f1* and *Nanog*), naive state-related genes (e.g., *Dnmlt3l* and *Alpg*),^22^^,^^60^ and differentiation-suppressive genes (e.g., *Rest* and *Hhla1*)^61^^,^^62^ (Figures 7C and S6D). *Cdh1* and *Tbx3* were no longer differentially expressed between A and B, though they were found to be broadly expressed across cluster 7, suggesting that they are necessary but insufficient by themselves for naive fate commitment. From these data, we propose that naive conversion requires not only naive inducer genes (e.g., *Cdh1* and *Tbx3*) but also the coordinated action of three gene populations: (1) pluripotency genes (e.g., *Pou5f1* and *Nanog*), (2) naive state-related genes (e.g., *Dnmlt3l* and *Alpg*), and (3) differentiation-suppressive genes (e.g., *Rest* and *Hhla1*). Furthermore, we found that several metallothionein (MT) family genes (e.g., *Mt2a*, *Mt1x*, *Mt1e*, *Mt1f*, *Mt1g*, and *Mt1h*) were markedly upregulated in subcluster A relative to subcluster B (Figure S7B). Although it is still unclear whether MTs are involved in the naive conversion process, elevated MT genes expression was observed in the naive state across AICAR- and CA-p38-induced cells as well as in VPA-induced naive cells (Figures S8A and S8B), implying that these genes may be included in naive state-related genes. In addition to these transcriptomic features, we further validated the naive conversion trajectory, using the Totem framework.^63^ After Totem preprocessing, we used multidimensional scaling (MDS) to obtain a two-dimensional embedding of the cells and overlaid the originally colored t-SNE-defined clusters onto this embedding (Figure 8A). Consistent with the original t-SNE map (Figure 6B), cluster 7, termed the “intermediate” state (red dots, Figure 8A), was mainly located between the primed state (clusters 0–3), the naive state (clusters 8 and 9), and the differentiated state (cluster 10). To explore cell-state transitions, we calculated cell-connectivity scores, which reflect the strength of cellular connectivity to nearby state, and found the highest connectivity in clusters 0–2, consistent with their identity as primed starting populations. Cluster 7 also exhibited an elevated connectivity score, supporting its role as an intermediate state (Figure 8B). Through a ranking based on the variance ratio criterion (VRC),^64^ we selected the one that scored highly and simultaneously resolved biologically meaningful populations (Figure 8C). This resulted in five Totem clusters: Totem cluster 1 (t-SNE clusters 0–2), primed state; Totem cluster 2 (t-SNE clusters 4–6), mesoderm-like state; Totem cluster 3 (t-SNE cluster 3), Oct4^+^/Nanog^+^ subpopulation; Totem cluster 4 (t-SNE clusters 8 and 9), naive state; and Totem cluster 5 (t-SNE cluster 10), ectoderm-enriched differentiated state. On the basis of this clustering, we reconstructed a minimum-spanning tree (MST), which revealed three principal trajectories: primed → naive via Totem cluster 3, primed → ectoderm, and primed → mesoderm (Figure 8C). Because an MST connects all clusters with the minimum number of links and avoids cycles,^65^^,^^66^ potential alternative routes such as the path from the “intermediate” state (t-SNE cluster 7) toward the ectoderm-enriched differentiated state (Totem cluster 5) are not represented, even though such transitions may exist biologically. To uncover potential alternative routes beyond those represented in the MST, we visualized connectivity scores as contour lines and a three-dimensional surface (Figures 8D and 8E). In these maps, cells in the “intermediate” state (t-SNE cluster 7) are broadly distributed along a high-connectivity corridor that extends in two directions: one toward the “naive” state (Totem cluster 4) and the other toward the ectoderm-enriched “differentiated” state (Totem cluster 5). This pattern suggests that cells departing from the primed state (Totem cluster 1) can pass through the intermediate state and branch toward either fate (Figure 8D). When the MST was overlaid on this surface, it showed a two-step route: cells first leave the primed state (Totem cluster 1) and enter the low-connectivity Oct4^+^/Nanog^+^ cluster (Totem cluster 3), then rise into the higher-connectivity intermediate state before reaching the naive state. The same high-connectivity region also extended toward the differentiated state, suggesting an additional plausible route (shown by the dashed line in Figure 8E) that links the intermediate state and the differentiated state. Many cluster 7 cells lie along the dashed line and the bold main line, indicating that the intermediate state can supply both naive and differentiated lineages. Combined with the t-SNE and RNA velocity results (Figure 7A), these findings indicate that the “intermediate” state serves as a transitional hub linking the primed state to divergent cell fates during naive conversion.

## Discussion

In this study, we demonstrated that activating the AMPK-p38 axis can convert primed hPSCs to the naive state under various conditions (PXGL, AXGY, AXG, and FXGA media). We demonstrated hallmark naive features of the converted cells, including the expression of naive markers, epigenomic resetting, increased mitochondrial activity, and cell-state dynamics during naive conversion. Our findings suggest that the AMPK-p38 axis would offer new insights for understanding and manipulating pluripotent status.

We previously showed that AMPK-p38 signaling is involved in maintaining and inducing the naive state of mouse ESCs,^23^^,^^25^ indicating its strong capacity to drive naive conversion in both mouse PSCs and hPSCs. We found that, in addition to PXGL, the AXGY, AXG, and FXGA also supported human naive conversion when combined with 1 mM AICAR or 5 mM metformin, both within AMPK-activating ranges (0.5–3 mM for AICAR and 1.25–10 mM for metformin)^67^^,^^68^ (Figures S1E and S1F), showing that AMPK activation is broadly effective across multiple naive maintenance conditions. Feeder layers (MEFs) remain essential in all known protocols for naive hPSCs and mouse PSCs, but their roles are unclear. Despite these shared features, certain culture requirements differ between species. For instance, Ndiff227 medium suited to human PSCs in our system but not mouse cells, whereas serum was indispensable for mouse naive conversion under AMPK-p38 activation but unhelpful for human PSCs. Once converted, mouse naive cells can grow feeder free in 2iL,^23^ whereas our human naive cells developed karyotype abnormalities after prolonged feeder-free culture in PXGL (data not shown). PXGL contains an MEK inhibitor, and prolonged MEK inhibition has been reported to induce genomic instability in naive hPSCs.^22^^,^^69^ However, it remains unclear whether the observed abnormalities result from MEK inhibition or from AICAR-mediated conversion. Further optimization, including the development of MEK inhibitor-free induction and maintenance conditions, will be required to achieve stable long-term feeder-free culture of naive hPSCs and to clarify the origin of these abnormalities.

To clarify the role of AMPK-p38 signaling in the context of existing naive conversion approaches, we compared our findings with those of previously reported methods. For example, human ESC naive conversion under 3iL (2i plus a BMP/AMPK inhibitor, dorsomorphin)^16^ relies on a broad inhibitor affecting both BMP and AMPK, leaving it unclear whether AMPK inhibition is the key. Moreover, that study used mTeSR as the basal medium, where we failed to induce naive conversion with AICAR (data not shown), suggesting that the distinct basal conditions complicate direct comparison. Beyond differences in basal conditions, controversy has also surrounded the role of p38 in naive conversion. For instance, naive human stem cell medium (NHSM)^15^ required SB203580, a p38 inhibitor, apparently contradicting our findings. However, our CA-p38-induced cells, together with other naive PSCs (e.g., Reset cells^31^ and chemically Reset cells^19^), closely resemble genuine HNES1,^48^ whereas NHSM-derived cells group with primed cells (Figure 5B). Consistent with this, NHSM was later updated to human enhanced naive stem cell medium (HENSM),^21^ removing the need for p38 inhibition. In contrast, in FINE cells,^20^ a p38 activator (dasatinib) was required. Notably, adding the p38 inhibitor SB203580 reduced naive induction efficiency in the FXGAY/A protocol (Figure S2A), suggesting that p38 activity is involved not only in our system but also in other methods. Taken together, these findings support the conclusion that p38 is a critical mediator of naive conversion induced by AMPK activators.

Another well-known downstream target of AMPK is mTOR inhibition.^70^ Indeed, the mTOR inhibitor Torin1 was reported to induce naive hPSCs,^9^ but we could not replicate these findings with rapamycin (data not shown). Interestingly, key naive genes were reported to remain low in Torin1-treated hPSCs,^9^ whereas they were found to be strongly upregulated under our AMPK-p38 activation conditions (Figure 5A), suggesting that under our conditions, AMPK-p38 signaling, rather than mTOR inhibition, was the primary driver of naive conversion. Typically, human naive conversion requires multiple genes and growth factors plus basal naive media^9^^,^^15^^,^^16^^,^^17^^,^^18^^,^^19^^,^^20^^,^^21^^,^^22^^,^^31^ (Table S1), making it challenging to pinpoint a core mechanism. VPA, an HDAC inhibitor, has been reported as a single-component approach,^19^ but it broadly affects epigenetics. By contrast, our study shows that simply activating AMPK-p38 under naive-supportive conditions is sufficient to induce human naive conversion, helping clarify the pivotal pathway involved.

We, therefore, used this simple induction system to investigate the mechanisms underlying the naive conversion process by performing scRNA-seq to capture the dynamics of state transitions. RNA velocity analysis (Figure 7A) suggested that t-SNE cluster 7 serves as an intermediate between the primed and naive states, with trajectories diverging toward either naive conversion or neural differentiation. This conclusion was independently supported by the Totem framework (Figures 8A and 8B), which identified cluster 7 as a highly connected state bridging the primed cells to both fates. To explore this population further, we re-clustered t-SNE clusters 7–11 and identified two subpopulations, subclusters A and B, that share broad transcriptional profiles but differ in only a few critical genes dictating naive (A) versus differentiated (B) outcomes (Figure 7B). The high connectivity scores assigned to cluster 7 reinforce the view that it represents a plastic and unstable state, in which even minor transcriptional imbalances can shift cells toward distinct fates. The presence of such a plastic, unstable intermediate state during naive conversion may help explain why small differences in culture conditions or induction protocols can yield divergent outcomes across naive-conversion methods. Successful progression toward the naive identity appears to depend on the coordinated action of three gene categories: (1) pluripotency genes, (2) naive state-related genes, and (3) differentiation-suppressive genes. Another interesting finding of ours is that multiple metallothionein (MT) family genes (e.g., *Mt1f*, *Mt1e*, *Mt2a*, *Mt1x*, *Mt1g*, and *Mt1h*) were markedly upregulated in subcluster A compared with subcluster B (Figure S7B). MTs are zinc (Zn)-binding proteins that mitigate oxidative stress and safeguard genomic integrity by suppressing mitochondrial reactive oxygen species (ROS).^71^^,^^72^ These functions have been implicated in longevity, as MTs help limit oxidative damage and maintain cellular homeostasis.^73^^,^^74^ In addition, Zn signaling has been shown to regulate pluripotency, with MT genes upregulated by Zn in hPSCs.^75^ Although the precise intersection between AMPK and MT pathways remains unclear, our findings raise the possibility that MTs act downstream of AMPK to connect naive state regulation with anti-aging processes. In this context, it is notable that AMPK itself is widely recognized as a central regulator of aging and metabolism, and the AMPK activators used in our system are also well established in aging research. For example, AICAR has been reported to attenuate cellular senescence,^76^ enhance cognitive function in aged mice,^77^ and promote skeletal muscle regeneration.^78^ Another AMPK activator, metformin, which also induced naive conversion in our study, has been implicated in anti-aging and is under clinical investigation for its association with longevity.^79^^,^^80^^,^^81^ Furthermore, pluripotency itself has been increasingly linked to anti-aging and rejuvenation pathways. Ectopic expression of pluripotency genes can restore youthful transcriptional signatures^82^ and mitigate aging-related phenotypes,^83^^,^^84^ largely through epigenetic reprogramming involving DNA demethylation. As the naive state in early embryos is characterized by minimal DNA methylation,^13^^,^^85^ naive conversion accompanied by epigenetic resetting could represent a promising strategy for rejuvenation. Together, these observations suggest that AMPK-p38 activation not only provides a straightforward and efficient route to naive pluripotency but may also intersect with molecular programs relevant to aging and longevity.

### Limitations of the study

This study has several limitations. First, the conversion experiments were performed in a limited number of hPSC lines. Second, although the converted cells exhibited multiple features consistent with naive pluripotency, further functional analyses will be needed to more fully define their developmental potential and long-term stability. Third, while our data identify p38 as a key downstream mediator of AMPK signaling during naive conversion, the molecular mechanisms linking AMPK activation to p38 and downstream effectors remain to be clarified. Fourth, naive conversion in our system still depended on feeder cells, and the contribution of feeder-derived signals to induction and maintenance of the naive state remains unresolved. Finally, karyotypic abnormalities were observed in some naive PSC cultures. Because PXGL contains an MEK inhibitor, and prolonged MEK inhibition has been associated with genomic instability in naive hPSCs, it remains unclear whether these abnormalities arose during maintenance or during the initial AICAR-mediated conversion step. Further optimization of induction and maintenance conditions will be important for achieving more stable long-term culture of naive hPSCs.

## Resource availability

### Lead contact

Further information and requests for resources and reagents should be directed to and will be fulfilled by the lead contact, Jun Yamashita (juny@m.u-tokyo.ac.jp).

### Materials availability

This study did not generate new unique reagents.

### Data and code availability

• Bulk RNA-seq data generated in this study are available in the Gene Expression Omnibus (GEO) database under accession number GEO: GSE198127. Single-cell RNA-seq data generated in this study are available in the GEO database under accession numbers GEO: GSE217008, GSE217009.
• This study did not generate new code.
• All other data supporting the findings of this study are available from the lead contact upon reasonable request.

## Acknowledgments

We are grateful to Dr. Yasuhiro Takashima (CiRA: Center for iPS Cell Research and Application, Kyoto, Japan) for valuable advice and discussion, and we thank Xinxiu Xu (The National Center for Drug Screening, Shanghai, China) for providing the plasmid of the constitutively active form of p38. This work was supported by a Japan Agency for Medical Research and Development (10.13039/100009619AMED) grant from Core Center for iPS Cell Research (JP21bm0104001), and Grants-in-Aid for Scientific Research on Innovative Areas (17H19676 and 19H05563) from 10.13039/501100001700Ministry of Education, Culture, Sports, Science and Technology (10.13039/501100001700MEXT), Japan.

## Author contributions

Z.Y. and J.Y. conceived the approach; Z.Y. designed and performed cell culture experiments; Y.L., A.H., and H.X. performed the EOS reporter experiments; J.Y. helped perform the RNA-seq experiments; W.F. carried out computational analysis of the RNA-seq data; Z.Y. and J.K.Y. wrote the paper; J.K.Y. supervised the study and acquired funding for this work.

## Declaration of interests

Department of Cellular and Tissue Communications, Graduate School of Medicine, The University of Tokyo, is an endowed department by TAKARA Bio. J.K.Y., Y.L., and Z.Y. are inventors on patent application WO 2022/191335 A1, filed by Kyoto University, related to methods for inducing naive PSCs using AMPK activators and p38 MAPK pathway activation.

## Declaration of generative AI and AI-assisted technologies in the writing process

The authors used AI-assisted tools (e.g., Chat GPT) for language refinement and editing. All scientific content, data analysis, and conclusions were independently developed and verified by the authors. The authors take full responsibility for the content of this manuscript.

## STAR★Methods

### Key resources table

**Table:** 

REAGENT or RESOURCE	SOURCE	IDENTIFIER
**Antibodies**		

Mouse anti-SUSD2 Antibody conjugated to PE (clone W5C5)	Bio Legend	Cat# 327406; RRID: AB_940654
Mouse anti-CD75 Antibody conjugated to Alexa 647 (clone ZB55)	BD Biosciences	Cat# 566350; RRID: AB_2869751
Mouse anti-CD57 Antibody conjugated to BV421 (clone NK-1)	BD Biosciences	Cat# 563896; RRID: AB_2632391
Mouse anti-Oct3-4 Antibody (clone C10)	Santa Cruz Biotechnology	Cat# sc-5279;RRID: AB_628051
Rabbit anti-NANOG Antibody (clone D73G4)	Cell Signaling Technology	Cat# 4903; RRID: AB_10559205
Mouse anti-NANOG Antibody (clone 23D2-3C6)	Thermo Fisher Scientific	Cat# MA1-017; RRID: AB_2536677
Rabbit anti-KLF17	Sigma	Cat# HPA024629; RRID: AB_1668927
Rabbit anti-TFE3	Sigma	Cat# HPA023881; RRID: AB_1857931
Rabbit anti-Thy1	Gene Tex	Cat# GTX130072; RRID: AB_
Mouse anti-PDGFR-β (clone PR7212)	R&D Systems	Cat# MAB1263; RRIS: AB_2886168
Goat anti-SOX17	R&D Systems	Cat# AF1924; RRID: AB_355060
Mouse anti-CXCR4 Antibody conjugated to PE (clone 12G5)	Bio Legend	Cat# 306506; RRID: AB_314612
Rabbit anti-MAP2	Cell Signaling Technology	Cat# 4542; RRID: AB_10693782
Mouse anti-TUJ1 (clone 2G10)	Sigma	Cat# T8578; RRID: AB_1841228
Rabbit anti-H3K9me3	Active Motif	Cat# 39765; RRID: AB_2793334
Mouse anti-5mC (clone 33D3)	Active Motif	Cat# 39649; RRID: AB_2687950
Rabbit anti-5hmC	Active Motif	Cat# 39769; RRID: AB_10013602
Mouse-GFP (clone 3E6)	Thermo Fisher Scientific	Cat# A-11120; RRID: AB_221568
PE anti-mouse (MHC) H-2 (clone M1/42)	Bio Legend	Cat# 125506; RRID: AB_1227705

**Chemicals, peptides, and recombinant protein**		

Recombinant human LIF	FUJIFILM Wako	Cat# 121-06663
Recombinant human LIF	Gibco	Cat# PHC9481
Recombinant human Activin A	R&D Systems	Cat# 338-AC-010
Recombinant human bFGF	FUJIFILM Wako	Cat# 068-04544
Recombinant human Noggin	R&D Systems	Cat# 6057-NG
Recombinant human Wnt-3a	Proteintech	Cat# HZ-1296
AICAR	FUJIFILM Wako	Cat# 011-22533
Metformin Hydrochloride	FUJIFILM Wako	Cat# 136-18662
Valproic acid sodium salt	Sigma	Cat# P4543
PD0325901	Sigma	Cat# PZ0162
AZ628	Selleckchem	Cat# S2746
XAV939	Millipore	Cat# 575545
Gӧ6983	FUJIFILM Wako	Cat# 078-06441
Y-27632	FUJIFILM Wako	Cat# 034-24024
SB203580	FUJIFILM Wako	Cat# 199-16551
SB431452	Tocris	Cat# 1614
DAPI	Invitrogen	Cat# D1306
Doxycycline	FUJIFILM Wako	CAS RN: 10592-13-9
Penicillin	Meiji	Cat# 4987-222-63767-1
Streptomycin	Meiji	Cat# 4987-222-66564-3
G418	Nacalai Tesque	CAS RN: 108321-42-2
Puromycin	Nacalai Tesque	CAS RN: 58-58-2

**Deposited data**		

RNA-seq data	This paper	GEO: GSE198127
Single-cell RNA-seq data	This paper	GEO: GSE217008, GSE217009

**Recombinant DNA**		

PB-EOS-C(3+)-EiP (EGFP-IRES-Puro)	Hotta et al., 2009^32^	N/A
tetracycline-inducible (Tet-ON) constitutive active form of p38	Xu et al., 2013^47^	N/A

**Critical commercial assays**		

SYBR Green PCR Master Mix	Applied Biosystems	Cat# 4367659
RNeasy Mini Kit	QIAGEN	Cat# 74104
RNase-Free DNase Set (50)	QIAGEN	Cat# 79254
SuperScript First-Strand Synthesis SuperMix	Invitrogen	Cat# 11752-050
SuperScript™ II Reverse Transcriptase 10,000 units	Invitrogen	Cat# 18064014
RNA Clean & Concentrator-5 kit	Zymo Research	Cat# R1013
NEBNext® Poly(A) mRNA Magnetic Isolation Module	New England BioLabs	Cat# E7490S
SMART-Seq® Stranded Kit	Takara Bio	Cat# 634442
Novaseq SP 200 cycles kit v1.5	Illumina	Cat# 20040719
BD Rhapsody Targeted mRNA and Abseq Reagent kit	BD Biosciences	633771
KAPA Hifi HS readymix	NIPPON Genetics	KK2602
AmPure XP	Beckman Coulter	A63881
NEBNext Ultra II FS Library Prep Kit	New England Biolabs	E7805L
KAPA library quantification kit for illumina	NIPPON Genetics	KK4873/D
Tetramethylrhodamine, Ethyl Ester, Perchlorate (TMRE)	Life Technology	Cat# T669

**Oligonucleotides**		

Primers for qPCR, see Table S2	This paper	N/A

**Software and algorithms**		

ImageJ	ImageJ	https://imagej.nih.gov/ij/

### Experimental model and study participant details

#### Cell lines

Primed H9 human ESCs were obtained from WiCell (WA09).

The primed human iPSC lines Ff-I14 and 1231A3 were provided by the Center for iPS Cell Research and Application (CiRA), Kyoto University. H9 ESCs were authenticated by WiCell via karyotyping and STR analysis prior to shipment and were confirmed to be mycoplasma-negative. The iPSC lines Ff-I14 and 1231A3 were authenticated by CiRA using STR profiling, and mycoplasma contamination testing was performed by CiRA prior to distribution. All cell lines used in this study were confirmed to be mycoplasma-negative.

### Method details

#### Cell culture

hiPSC lines (Ff-I14 and 1231A3) were provided by the Center for iPS Cell Research and Application, Kyoto University, and the hESC line (H9) was provided by WiCELL. Primed PSCs were maintained on Matrigel (Invitrogen, 1:60 dilution)-coated dishes in StemFit AK02N (Ajinomoto).^86^ Cells were passaged every 3–7 days as single cells using TrypLE™ Select CTS™ (Gibco), with 10 μM Y27632 (Fujifilm) added for the first 24 hr post-passage. Naïve human PSCs were maintained in PXGL or FXGL media on Matrigel-coated 6-well plates with mitomycin C–inactivated mouse embryonic fibroblasts (MEFs). PXGL is the Ndiff227 medium (Takara Bio) supplemented with PD0325901 (1 μM, Sigma), XAV939 (2 μM, Millipore), Go6983 (2 μM, Fujifilm),^24^ human LIF (10 ng/ml, Fujifilm), and penicillin/streptomycin (Meiji). FXGL consists of Ndiff227 supplemented with FGFR1 inhibitor PD166866 (1 μM, Tocris), XAV939 (2 μM, Millipore), Go6983 (2 μM, Fujifilm), human LIF (10 ng/ml, Fujifilm), and penicillin/streptomycin. Cells were passaged on MEF feeders every 5–10 days as single cells (TrypLE™ Select CTS™, Gibco), again using 10 μM Y27632 for the first 24 hr. Medium was changed daily. For naïve induction, AICAR (1 mM, Fujifilm) was added to PXGL, AXGY, or AXG media,^23^^,^^25^ while metformin was added to AXGY and AXG. AXGY and AXG are also based on Ndiff227 (Takara Bio), further supplemented with AZ628 (5 μM, Selleckchem), XAV939 (2 μM, Millipore), Go6983 (2 μM, Fujifilm), and Y27632 (10 μM, Fujifilm) for AXGY,^22^ while AXG lacks Y27632. VPA^19^ was added to Ndiff227 basal medium together with PD0325901 (1 μM, Sigma), human LIF (10 ng/ml, Fujifilm), and penicillin/streptomycin for naïve induction. As a comparison with previously reported induction methods, FXGAY/A medium^22^ was also tested. FXGAY/A consists of FGFR1 inhibitor PD166866 (1 μM, Tocris), XAV939 (2 μM, Millipore), Go6983 (2 μM, Fujifilm), AZ628 (5 μM, Selleckchem), Y27632 (10 μM, Fujifilm), and Activin A (10 ng/ml, Peprotech). For feeder-free culture, naïve human PSCs were seeded onto Matrigel-coated 6-well plates in PXGL medium with 10 μM Y27632 (Fujifilm) and laminin-511 (iMatrix-511 silk; Nippi) for the first 24 hr. Thereafter, the medium was replaced daily with fresh PXGL.

#### EOS-GFP and CA-p38 transfection

Mouse ES cells and iPS cells that are in the naïve state, and naïve state human ES cells and iPS cells have already been reported to preferentially use the distal enhancer (DE) in Oct4 transcription,^2^^,^^17^^,^^31^^,^^87^ while mouse EpiSCs, human ES cells and iPS cells, known as primed state cells, preferentially use the proximal enhancer (PE).^15^^,^^88^ PB-EOS-C(3+)-EiP (EGFP-IRES-Puro) has been described as a reporter of DE-Oct4 transcription and is used as a naïve state marker.^31^^,^^32^ To monitor the transition between naïve and primed states in human PSCs, we introduced the PB-EOS-C(3+)-EiP piggyBac vector and pHL-EF1a-hcPBase-iC-A into H9 ESCs (H9-EOS) and Ff-I14 iPSCs (Ff-I14-EOS). 24 hr after electroporation by NEPA21 (Nepagene), cells were selected by treatment of puromycin (Nacalai, 10 μg/ml) for 5 days. Transfected cells were maintained on Matrigel-coated dishes in StemFit AK02N (Ajinomoto).

To generate CA-p38 H9-EOS lines, H9-EOS cells were transfected with a tetracycline-inducible (Tet-ON) construct encoding a constitutively active form of p38 (CA-p38), featuring D176A/F327S mutations in p38 cDNA within a piggyBac (PB) vector co-expressing rtTA and mCherry.^25^^,^^47^ Doxycycline (DOX) treatment induced mCherry expression, and mCherry^+^ cells were purified by FACS. Transfected cells were maintained on Matrigel-coated dishes in StemFit AK02N (Ajinomoto).

#### Flow cytometry

Cells were dissociated into single cells with TrypLE™ Select CTS™ and stained with conjugated antibodies and 4', 6-diamidino-2-phenylindole (DAPI).

SUSD2 (Clone W5C5, SUSD2-PE, BioLegend 327406), CD75/CD75s (Clone ZB55, CD75-APC, BD 566350), and CD57 (Clone NK-1, CD57-BV421, BD 563896) were used for flow cytometry.^24^^,^^33^

#### Immunostaining

Immunostaining was performed as described.^89^ Cells were fixed in 4% paraformaldehyde for 15 min, then blocked for 1 hr at room temperature in Blocking One Histo (Nacalai, 1:20) / PBS + 0.5% Triton. Primary antibodies diluted in PBS + 0.5% Triton were incubated overnight at 4°C. Secondary antibodies (anti-rabbit, anti-mouse, anti-goat, Alexa488/Alexa546 conjugates, Thermo) in PBS + 0.5% Triton were applied for 1 hr at RT. After washing with PBS/Tween-20, nuclei were counterstained with DAPI.

For methylated DNA staining (5mC and 5hmC), fixed cells were permeabilized with PBS containing 0.5% Triton X-100 for 1 hr, treated with 2 N HCl for 30 min at room temperature to denature DNA, and neutralized. Samples were then subjected to immunostaining with primary antibodies overnight at 4°C, followed by incubation with appropriate secondary antibodies for 1 hr at room temperature. After washing, nuclei were counterstained with DAPI.

#### RNA FISH

RNA FISH was performed using BAC clones RP11-13M9 (XIST), RP11-256P2 (UTX), and RP11-155O24 (HUWE1), labeled with Green, Cy3, and Cy5, respectively. Cells were treated with 0.2 N HCl for 20 min at room temperature and 0.2% Triton X-100 in PBS for 10 min. Samples were digested with 0.005% pepsin in 0.1 N HCl at 37°C (40–41 min for primed cells and 2–6 min for naïve cells), washed, and air-dried. Probe solution was denatured at 85°C for 10 min, applied to samples, and hybridized overnight at 37°C. After washing in 50% formamide/2×SSC and 1×SSC at 37°C, nuclei were counterstained with DAPI and mounted with antifade reagent.

#### Western blotting

CA-p38 H9-EOS cells were lysed in sample buffer containing 2-mercaptoethanol (ME, Nacalai). Proteins were resolved on a BlotTM Gel (Invitrogen) and transferred to nitrocellulose membranes.

Membranes were blocked with Blocking One (Nacalai) for 30 min, then incubated overnight at 4°C with primary antibodies against p38 (Cell Signaling 9212S, 1:1000), phosphorylated p38 (Thr180/Tyr182, Cell Signaling 9215S, 1:1000), or β-actin (Sigma A5441, 1:10000), all diluted in Can Get Signal Immunoreaction Enhancer (Toyobo).

Horseradish peroxidase (HRP)-conjugated anti-mouse or anti-rabbit IgG antibodies (Cell Signaling, 1:1000–1:3000), diluted in the same enhancer solution, were used as secondary antibodies and incubated for 2 hours at room temperature (RT). Signals were detected using the Immobilon Western chemiluminescent substrate (Millipore).

#### RNA isolation and quantitative reverse transcription polymerase chain reaction (RT-qPCR)

Total RNA was extracted using the RNeasy Mini kit (Qiagen). cDNA was synthesized with SuperScript III (Invitrogen). All qPCR reactions were performed in technical triplicates using SYBR Green Master Mix (Applied Biosystems), and expression levels were normalized to RPS18.

#### Mitochondrial activity

Mitochondrial membrane potential was assessed by staining cells with 20 nM TMRE (Thermo Fisher) in culture medium for 10 min at 37°C, followed by flow cytometry using the PE channel on a CytoFLEX flow cytometer (Beckman Coulter) or confocal microscopy.^31^

#### RNA-sequencing

RNA-seq was performed on primed H9-EOS, primed CA-p38 H9-EOS, AICAR induced naïve H9-EOS, CA-p38 induced naïve CA-p38 H9-EOS, and VPA induced naïve H9-EOS.

For sample preparation, primed state cells were dissociated by TrypLE™ Select CTS™, and CD75^+^/SUSD2^+^ naïve state cells were sorted using FACS. Total RNA was isolated by using the RNeasy Mini kit (Qiagen) and purified with the RNA Clean & Concentrator-5 kit (Zymo Research) according to the manufacturer's instructions. Polyadenylated RNA (polyA) was enriched by using NEBNext® Poly(A) mRNA Magnetic Isolation Module (New England BioLabs). RNA-seq libraries were generated by using SMART-Seq® Stranded Kit (TaKaRa bio), and sequenced by Novaseq 6000. Reads were aligned to human genome build GRCh38.p13 and Reads Per Kilobase of transcript, per Million mapped reads (RPKM) calculation were performed by using RSEM (RNA-Seq by Expectation-Maximization).

Comparative datasets were obtained from the European Nucleotide Archive (ENA): ERP006823,^31^ SRP059279,^90^ SRP045911,^49^ SRP055810,^91^ SRP074076.^50^ All 29 fastq files are adapter trimmed by cutadapt-1.15 using trim_galore-0.4.4_dev with –stringency 3 option, and then mapped to Ensembl GRCh38 release 100 reference cDNA and ncRNA sequences using Bow-tie2 v2.2.5 with the very-sensitive-local option. Among a total of 1,800,071,774 (single or paired) reads, 1,254,839,844 (69.7%) were successfully mapped and quantified as genes with a threshold of MAPQ score ≥ 1. A total of 61,222 genes (43,813 on average) were detected for 29 samples. The following analyses were performed using R ver. 4.0.3 after non-categorized limma voom normalization.^92^ A total of 25 samples from naïve hESCs, primed hESCs, AICAR- and CA-p38 induced naïve hPSCs were analyzed. The heatmap (Figure 5A) was analyzed for a selected gene set of pluripotency regulators, naïve and primed state associated genes^9^^,^^17^^,^^19^^,^^31^^,^^48^ as depicted.

#### Single-cell RNA-sequencing

We collected 8 different samples for single-cell RNA-seq analysis, including primed H9-EOS (hESCs), cells treated with AICAR+PXGL for 2 weeks (AICAR 2 weeks) or 4 weeks (AICAR 4 weeks), and AICAR-induced naïve hESCs cultured feeder-free for more than 10 passages (long-cultured naïve H9-EOS). The cell number of each sample was counted using Flow Count Fluorospheres (Beckman Coulter) and staining with anti-human Hashtag-A antibodies (BioLegend, clones 7–15).

In total, 36,664 cells were pooled into a single tube and immediately processed for cDNA trapping and reverse transcription using a BD Rhapsody system, following the manufacturer’s instructions. The BD Rhapsody scanner detected 26,318 cells captured (one bead per cell).

The resulting cDNA and hashtag libraries were amplified by TAS-Seq^93^ and sequenced by ImmunoGeneTeqs on an Illumina NovaSeq with an SP 200 cycle kit v1.5 (67 bp for read1, 155 bp for read2).

Adapter trimming of sequencing data was performed by using cutadapt 2.10. Filtered reads were chunked to 16 parts for parallel processing by using Seqkit 0.9.0. Filtered cell barcode reads were annotated by Python script provided by BD with minor modification for compatible to Python3.7, associated cDNA reads were mapped to Ensembl cDNA and ncRNA (build GRCh38, release-101) with reporter gene EGFP-IRES2-Puromycin-3'UTR by using Bowtie2-2.3.4.1 by the following parameters: -N 1 - -very-sensitive-local --seed 656565 --reorder, and associated Hashtag reads were mapped to Known barcode sequence+BAAAAA by using Bowtie2-2.4.2 by the following parameters:-D 50 -R 20 -N 0 -L 8 -i S,1,0.75 --norc --seed 656565 --reorder --trim-to 3:21 --score-min L,-9,0 --mp 3,3 --np 3 --rdg 3,3. Then, cell barcode information of each read was added to the bowtie2-mapped BAM files, and read counts of same gene symbols of each cell barcode were counted. Inflection point of the knee-plot of cell barcode/read number was detected by using DropletUtils package in R 3.5.3, and cell barcodes below the inflection threshold were filtered out. Each hashtag read was normalized by total read counts into the smallest total read counts linearly and each cell were annotated to each hashtag based on fold-change between first- and second-most highly counted hashtags. Then, cells were ordered ascending by the fold-change, and top 3.82 % cells were filtered out as doublet cells (probability of the % of doublet was estimated by Poisson's distribution calculated by load cell number and total number of Rhapsody wells). Distribution-based error correction for background subtraction of gene-expression matrix was performed as described previously^93^ with the following parameters (maximum expression of log2(x+1) was over 8, maximum average expression of first component was over 5.5, and minimum difference of average expression between first- and second-components was over 5). We then analyzed cells assigned to hashtag 7-10 for this work.

In Figure 6B, a total of 3,314 out of 12,033 cells after quality check were normalized and analyzed by Seurat package version 4.0.2.^94^ in R version 4.0.5. Twelve clusters (cluster 0-11) were obtained with 2000 most highly variable genes under the parameters of PCA dimension=37, k.param=50, and resolution=2.0, and two-dimensional t-SNE is drawn by the package. In Figure 7A, we performed RNA velocity analysis as follows. After adapter trimming, quality filtering raw scRNA-seq fastq data, cell barcode reads were annotated by Python script provided by BD with minor modification for compatible to Python3.7. Associated cDNA reads were mapped to reference genome (build GRCh38 release-101 with reporter gene) by using HISAT2-2.2.1.^95^ by the following parameters: -q -p 6 –rna-strandness F –very-sensitive –seed 656565 –reorder –omit-sec-seq –mm. For HISAT2 index build, Corresponded ensembl gtf file was filtered to retain protein-coded RNA, long non-coding RNA and T cell chain/immunoglobulin chain annotations according to 10X Genomics's method (https://support.10xgenomics.com/single-cell-gene-expression/software/pipelines/latest/advanced/references#mkgtf). Addition of reporter gene (EGFP-IRES2-Puromycin-3'UTR) into reference genome and GTF files was performed according to 10X Genomics's method (https://support.10xgenomics.com/single-cell-gene-expression/software/pipelines/latest/using/tutorial_mr, “Add a Marker Gene to the FASTA and GTF” section). Then, cell barcode information of each read were added to the HISAT2-mapped BAM files, and associated gene annotations were assigned by using featureCounts v2.0.2 by following parameters: -T 2 -Q 0 -s 1 -t gene -g gene_name –primary -M -O –largestOverlap –fraction -R BAM. In featureCounts analysis, “gene” annotation was used for capturing un-spliced RNA information for RNA velocity analysis, and primary annotations were kept. Then, resulted BAM file was splitted by valid cell barcodes by using nim 1.0.6 and hts-nim v0.2.23. For each BAM file, we ran velocyto-0.17.17 by python-3.6.10 under anaconda3 environment. The resulted 3,314 loom files are combined into single loom file by loompy-3.0.6 package and imported into Seurat object. In Seurat, we used velocyto.R-0.6 library to calculate the velocity direction with kCells=100 on PCA space and draw the arrow embedding figure with n=50 on the original tSNE space. For Figure 7B, we first extracted cells of cluster 7,8,9, and 10 with tSNE_2 < -15 condition. Then we reassign clusters with higher resolution of 5 and obtained 26 clusters (A-Z). We analyzed differentially expressed genes by volcano plots between the cluster A and the cluster B for all of 37,273 genes and the results are drawn in Figure 7B by using ggplot2-3.3.3 package in R.

#### Trajectory analysis using totem

Single-cell trajectory analysis was carried out with the Totem workflow.^63^ Raw count matrices were normalised, low-quality cells and low-abundance genes were removed, and highly variable genes were retained. The filtered matrix was embedded by multidimensional scaling (MDS) and visualised in two dimensions. On this embedding, the CLARA k-medoids algorithm (k = 3–20) was run 10,000 times, each run yielding a distinct clustering. For every clustering, Mahalanobis-like inter-cluster distances were used to build a minimum-spanning tree (MST); the connectivity of each cluster (number of incident edges divided by the total number of clusters) was assigned to all cells in that cluster. Averaging these connectivity values across the 10,000 MSTs produced a single cell-connectivity score for every cell.

All clusterings were then ranked with the Calinski–Harabasz variance-ratio criterion (VRC) calculated on this one-dimensional connectivity vector. The top twenty VRC-scoring solutions were examined, and the clustering that clearly resolved three differentiation trajectories leading toward ectoderm, mesoderm, and the naïve state was selected. A new MST was rebuilt on this optimal clustering (distinct from the exploratory trees used for connectivity estimation); the cluster with the highest mean connectivity and enriched for OCT4-positive primed cells was set as the root, and the tree was smoothed with Slingshot^96^ to generate continuous lineage curves and pseudotime values.

#### *In vitro* differentiation

Naïve cells were “re-primed” before *in vitro* differentiation by passaging them on Matrigel-coated dishes in StemFit AK02N (Ajinomoto) for ∼1 month.

For endoderm differentiation, the medium was switched to mTeSR1 (STEMCELL Technologies) from StemFit AK02N (Ajinomoto), and cultured re-primed cells for 1 week. Then mTeSR (STEMCELL Technologies) medium was switched to RPMI1640 (Gibco)+B27 medium (RPMI1640, 2 mM L-glutamine, x1 B27) with Activin A (R&D, 100 ng/ml) and Wnt3A (Proteintech, 25 ng/ml).

The next day, the medium was changed to RPMI medium with Activin A (R&D, 100 ng/ml) and 0.2% serum.

For mesoderm differentiation, re-primed cells were cultured in StemFit AK02N (Ajinomoto) up to a confluence state, and then cells were covered with Matrigel (Invitrogen, 1:60 dilution) diluted in mouse embryonic fibroblast conditioned medium (MEF-CM) supplemented with hbFGF (Fujifilm,4 ng/ml) for 1 day. On the following day, MEF-CM was replaced with RPMI1640 (Gibco)+B27 medium (RPMI1640, 2 mM L-glutamine, x1 B27 supplement without insulin) supplemented with Activin A (R&D, 100 ng/ml) for 24 hr, followed by human Bone morphogenetic protein 4 (R&D, 10 ng/ml) and hbFGF (Fujifilm,10 ng/ml) for 4 days with no culture medium replacement. Finally, the medium was switched to RPMI1640 (Gibco)+B27 medium (RPMI1640, 2 mM L-glutamine, x1 B27), and cultured for 1 week.

For ectoderm differentiation, re-primed cells were cultured with Ndiff227 (Takara Bio) supplemented with hbFGF (Fujifilm,10 ng/ml), SB431542 (Tocris, 20 μM), and Noggin (R&D, 260 ng/ml) for 4 days,^14^ and then the medium was switched to Ndiff227 (Takara Bio) supplemented with hbFGF (Fujifilm,10 ng/ml) and SB431542 (Tocris, 20 μM).

#### Cell growth rate determination

Naïve cells maintained on MEF feeder cells were dissociated with TrypLE™ Select CTS™.

Dissociated cells were counted and stained with SUSD2-PE and CD75-APC.

The fraction of SUSD2^+^/CD75^+^ cells, as assessed by flow cytometry, was used to calculate the total number of naïve hPSCs.

### Quantification and statistical analysis

#### Imaging analysis

H3K9me3 fluorescent foci, global 5mC and 5hmC fluorescence intensity, and the intensity of Western blot bands were quantified using ImageJ. Fluorescence signals for H3K9me3, 5mC, and 5hmC were analyzed using ImageJ. Fluorescence intensity profiles were generated by drawing linear regions of interest across nuclei, and gray values along the indicated lines were used for quantitative assessment. For differentiation assays, marker-positive cells were manually counted relative to the total number of DAPI-positive nuclei within the captured image. Statistical analysis was performed only for Western blot data using a one-way repeated measures analysis of variance (ANOVA), followed by Tukey’s test as a post hoc comparison. For Western blot quantification, data are presented as mean ± SEM.

### Additional resources

This study did not generate additional resources.
