# Supplementary material for: AMPK-p38 axis converts human pluripotent stem cells to naive state
Source: iScience. 2026 Apr 1;29(5):115569. doi: 10.1016/j.isci.2026.115569 (PMC13122662; doi:10.1016/j.isci.2026.115569)
Supplement: Document S1. Figures S1–S8 and Tables S1 and S2 [file mmc1.pdf]

iScience, Volume *29*

Supplemental information

AMPK-p38 axis converts human pluripotent stem cells to naive state

Zhennan Yang, Yajing Liu, Huaigeng Xu, Junko Yamane, Akitsu Hotta, Wataru Fujibuchi, and Jun K. Yamashita

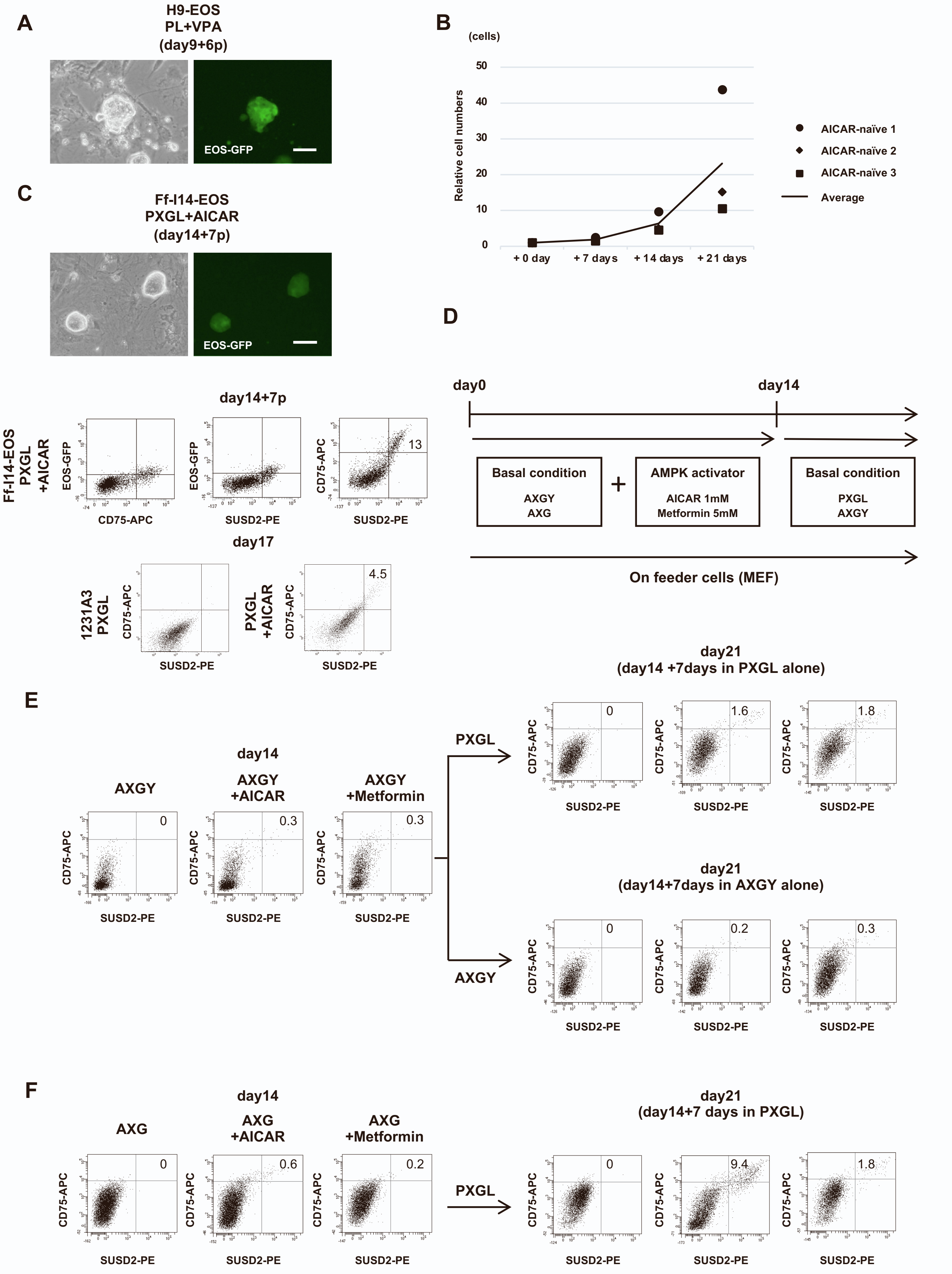
A
H9-EOS
PL+VPA
(day9+6p)
EOS-GFP
B
(cells)
Relative cell numbers
50
40
30
20
10
0
+ 0 day
+ 7 days
+ 14 days
+ 21 days
AICAR-naïve 1
AICAR-naïve 2
AICAR-naïve 3
Average
C
Ff-I14-EOS
PXGL+AICAR
(day14+7p)
EOS-GFP
day14+7p
Ff-I14-EOS
PXGL
+AICAR
EOS-GFP
CD75-APC
EOS-GFP
SUSD2-PE
CD75-APC
SUSD2-PE
13
day17
1231A3
PXGL
CD75-APC
SUSD2-PE
PXGL
+AICAR
CD75-APC
SUSD2-PE
4.5
D
day0
day14
Basal condition
AXGY
AXG
+
AMPK activator
AICAR 1mM
Metformin 5mM
Basal condition
PXGL
AXGY
On feeder cells (MEF)
E
day14
AXGY
AXGY
+AICAR
AXGY
+Metformin
CD75-APC
SUSD2-PE
0
0.3
0.3
PXGL
AXGY
day21
(day14 +7days in PXGL alone)
0
1.6
1.8
day21
(day14+7days in AXGY alone)
0
0.2
0.3
F
day14
AXG
AXG
+AICAR
AXG
+Metformin
0
0.6
0.2
PXGL
day21
(day14+7 days in PXGL)
0
9.4
1.8

Figure S1. Naïve Conversion of Human PSCs, Related to Figure 1.

(A) Images of naïve hESCs (H9-EOS) induced by VPA. VPA, valproic acid (day9+6p).
(B) Cell proliferation data of naïve hESCs (H9-EOS) in the PXGL on MEF feeder cells.
AICAR-naïve 1: day14+19p, AICAR-naïve 2: day14+20p, AICAR-naïve 3: day14+7p at day0 in figure.
(C) Naïve hiPSCs (Ff-I14-EOS and 1231A3) induced by AICAR treatment. Ff-I14-EOS exhibited dome-shaped colony morphology and strong EOS-GFP expression, confirming naïve state characteristics (Scale bar: 100 μm). Flow-cytometry analysis showed EOS-GFP, SUSD2, and CD75 expression in Ff-I14-EOS cells (day14+7p). For 1231A3 hiPSCs (day17), SUSD2 and CD75 expression was analyzed, demonstrating the presence of naïve marker–positive cell populations.
(D) Naïve conversion protocol using AICAR and Metformin. Both AICAR and Metformin are AMPK activators. AXGY; AZ628 (5 μM), XAV939 (2 μM), Go6983 (2 μM), and Y27632 (10 μM) in Ndiff227 medium.
(E) Flow-cytometry analysis of SUSD2 and CD75 expression (day14 AXGY basal condition and day14+7days cultured in PXGL and AXGY: day21).
(F) Flow-cytometry analysis of SUSD2 and CD75 expression (day14 AXG basal condition and day14+7days cultured in PXGL: day21).
Scale bars: 100 μm.

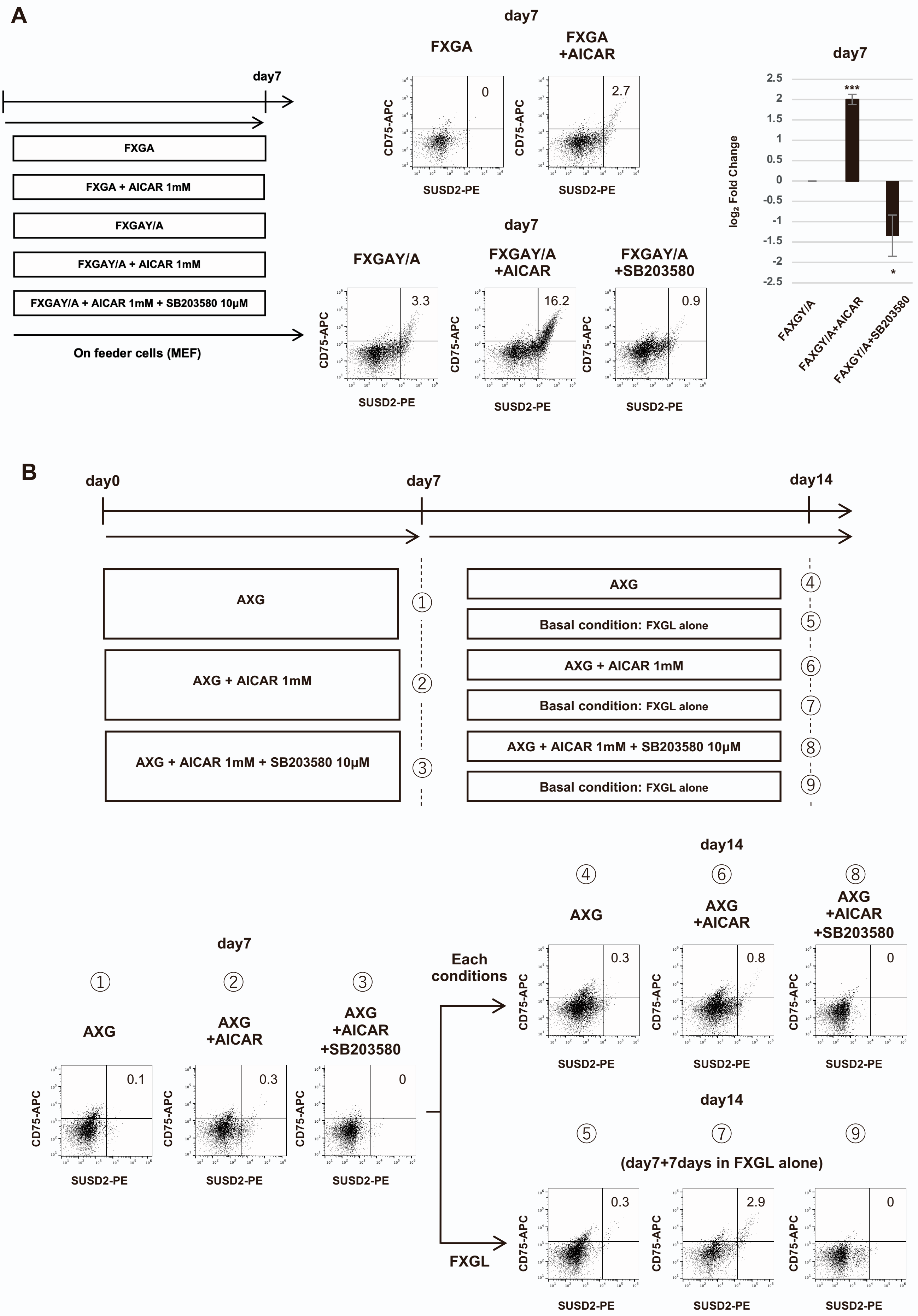

A
day7
FXGA
FXGA + AICAR 1mM
FXGAY/A
FXGAY/A + AICAR 1mM
FXGAY/A + AICAR 1mM + SB203580 10μM
On feeder cells (MEF)
day7
FXGA
FXGA +AICAR
0
2.7
CD75-APC
SUSD2-PE
day7
FXGAY/A
FXGAY/A +AICAR
FXGAY/A +SB203580
3.3
16.2
0.9
day7
log₂ Fold Change
***
*
FAXGY/A
FAXGY/A+AICAR
FAXGY/A+SB203580
B
day0
day7
day14
AXG
AXG + AICAR 1mM
AXG + AICAR 1mM + SB203580 10μM
Basal condition: FXGL alone
①
②
③
④
⑤
⑥
⑦
⑧
⑨
day7
AXG
AXG +AICAR
AXG +AICAR +SB203580
0.1
0.3
0
Each conditions
FXGL
day14
AXG
AXG +AICAR
AXG +AICAR +SB203580
0.3
0.8
0
day14
(day7+7days in FXGL alone)
0.3
2.9
0

Figure S2. Naïve Conversion Under Different Basal Conditions, Related to Figures 1 and 4.

(A) Naïve induction by AICAR (1 mM) under an FXGA basal condition (PD166866 1 μM, XAV939 2 μM, Gö6983 2 μM, AZ628 5 μM) was tested for suppression by SB203580 (10 μM), and to assess whether AICAR enhances induction under FXGAY/A (PD166866 1 μM, XAV939 2 μM, Gö6983 2 μM, AZ628 5 μM, Y-27632 10 μM, Activin A 10 ng/ml). Flow-cytometry analysis of SUSD2 and CD75 expression was performed on day 7 to compare basal conditions with naïve induction conditions. AICAR significantly increased induction efficiency by 4.1-fold compared to FXGAY/A alone (***$p < 0.01$), while SB203580 reduced it to 0.5-fold (*$p < 0.05$). *$p < 0.05$, ***$p < 0.01$ by one-way ANOVA followed by Dunnett's multiple-comparisons test ($n = 5$). Data are presented as mean ± SEM.
(B) Naïve conversion using AXG (a version of AXGY lacking Y-27632) supplemented with AICAR (1 mM), with or without the p38 inhibitor SB203580 (10 μM). Cells were cultured for 7 days (day 7) under these conditions, then for an additional 7 days (day 14) either under the same conditions or switched to FXGL medium. Flow-cytometry for SUSD2 and CD75 expression was performed on day 14 to evaluate naïve conversion efficiency.

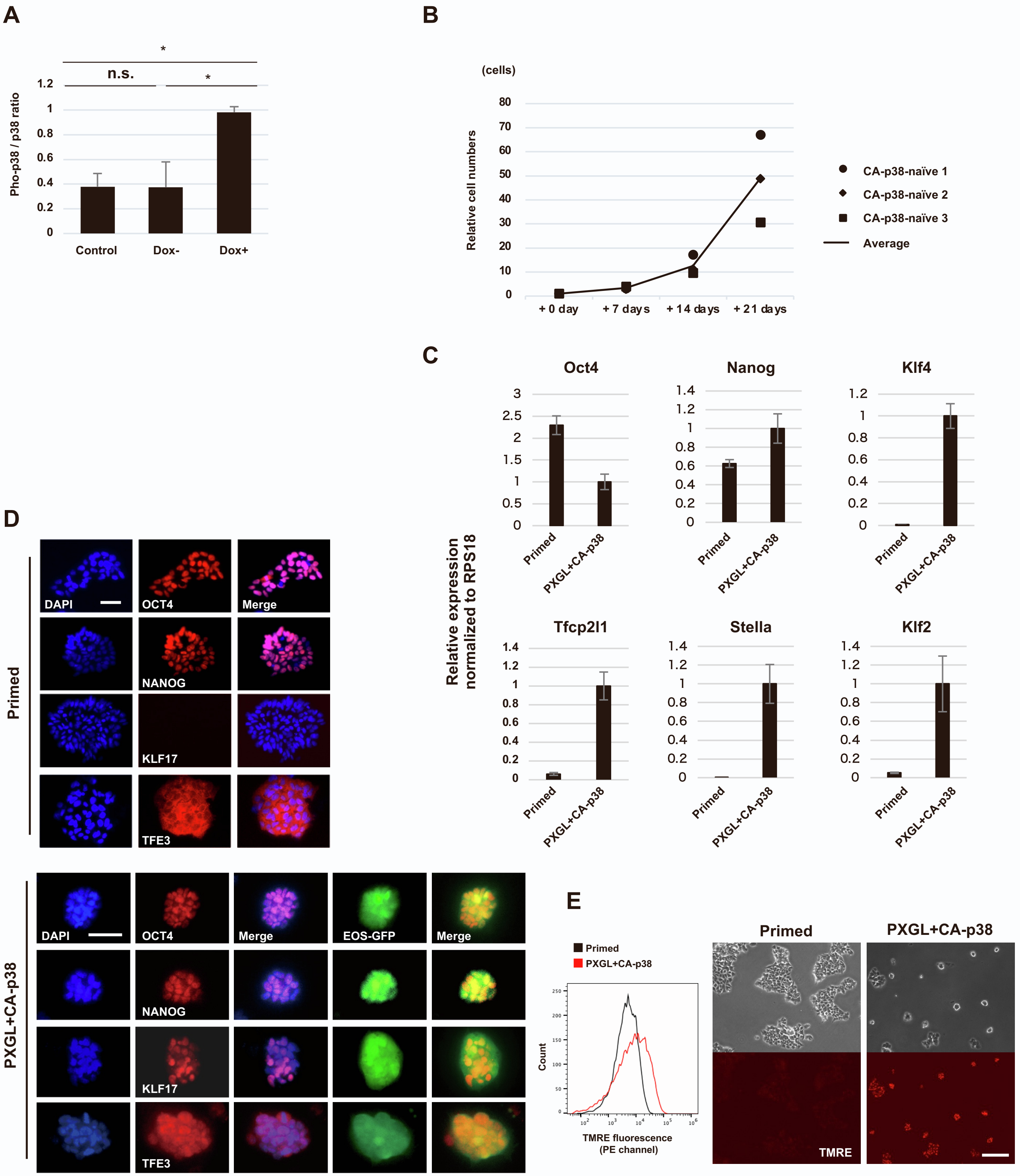

A
*
n.s.
*
Pho-p38 / p38 ratio
1.2
1
0.8
0.6
0.4
0.2
0
Control
Dox-
Dox+
B
(cells)
Relative cell numbers
80
70
60
50
40
30
20
10
0
+ 0 day
+ 7 days
+ 14 days
+ 21 days
CA-p38-naïve 1
CA-p38-naïve 2
CA-p38-naïve 3
Average
C
Relative expression normalized to RPS18
Oct4
Nanog
Klf4
Tfcp2l1
Stella
Klf2
Primed
PXGL+CA-p38
D
Primed
DAPI
OCT4
Merge
NANOG
KLF17
TFE3
PXGL+CA-p38
DAPI
OCT4
Merge
EOS-GFP
Merge
NANOG
KLF17
TFE3
E
Primed
PXGL+CA-p38
Count
TMRE fluorescence
(PE channel)
Primed
PXGL+CA-p38
TMRE

Figure S3. Naïve Reprogramming by CA-p38, Related to Figure 4.

(A) Western blot analysis of phosphorylated p38 (p-p38). β-Actin was used as a loading control. Error bars represent the standard error (SE) of three independent experiments ($n = 3$). $^*p < 0.05$.
(B) Cell proliferation data of naïve hESCs (CA-p38 H9-EOS) cultured in PXGL on MEF feeders. CA-p38-naïve 1 (day7+8p), CA-p38-naïve 2 (day7+16p), and CA-p38-naïve 3 (day7+10p) are set to day 0 in the figure.
(C) RT-qPCR analysis comparing sorted $SUSD2^+CD75^+$ naïve hESCs and conventional primed hESCs (CA-p38 H9-EOS). CA-p38: day7+10p. Error bars indicate the standard deviation (SD) of technical triplicates.
(D) Immunostaining for OCT4, NANOG, KLF17, and TFE3 in cells undergoing naïve conversion by CA-p38 (day7+12p).
(E) CA-p38–induced cells showed increased TMRE fluorescence as measured by both flow cytometry (PE channel) and fluorescence microscopy.
Scale bars: 50 μm in (D); 100 μm in (E). See also Table S2.

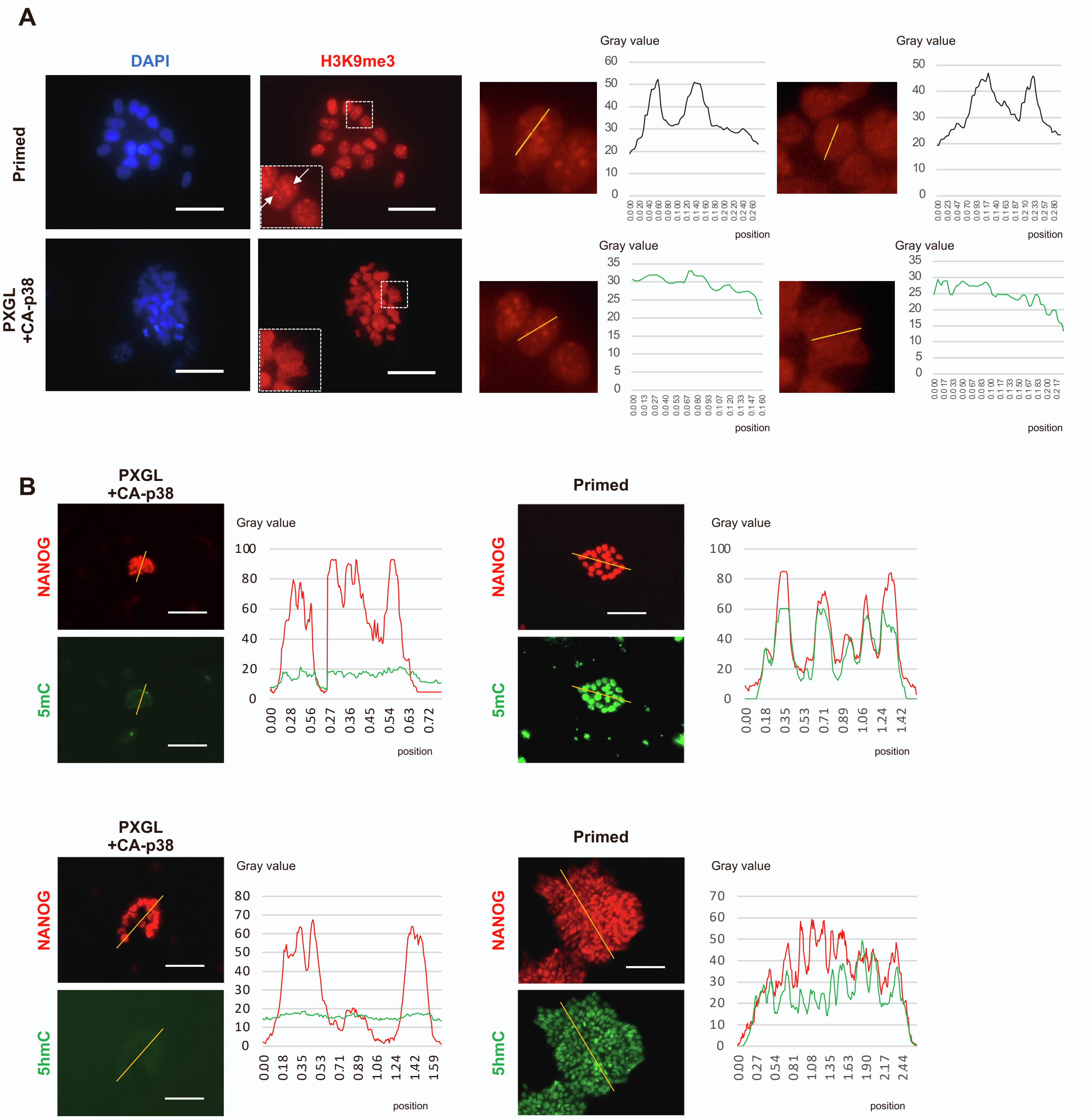

Figure S4. Epigenomic Analysis of CA-p38-Induced Naïve hPSCs, Related to Figure 3.

(A) Immunostaining of H3K9me3 with intensity and nuclear distribution profiles analyzed using ImageJ.
(B) Immunostaining for 5mC, 5hmC and NANOG. Quantification of global 5mC and 5hmC fluorescence intensity was performed using ImageJ.
Scale bars: 50 μm in A; 100 μm in B. CA-p38: day7+12p.

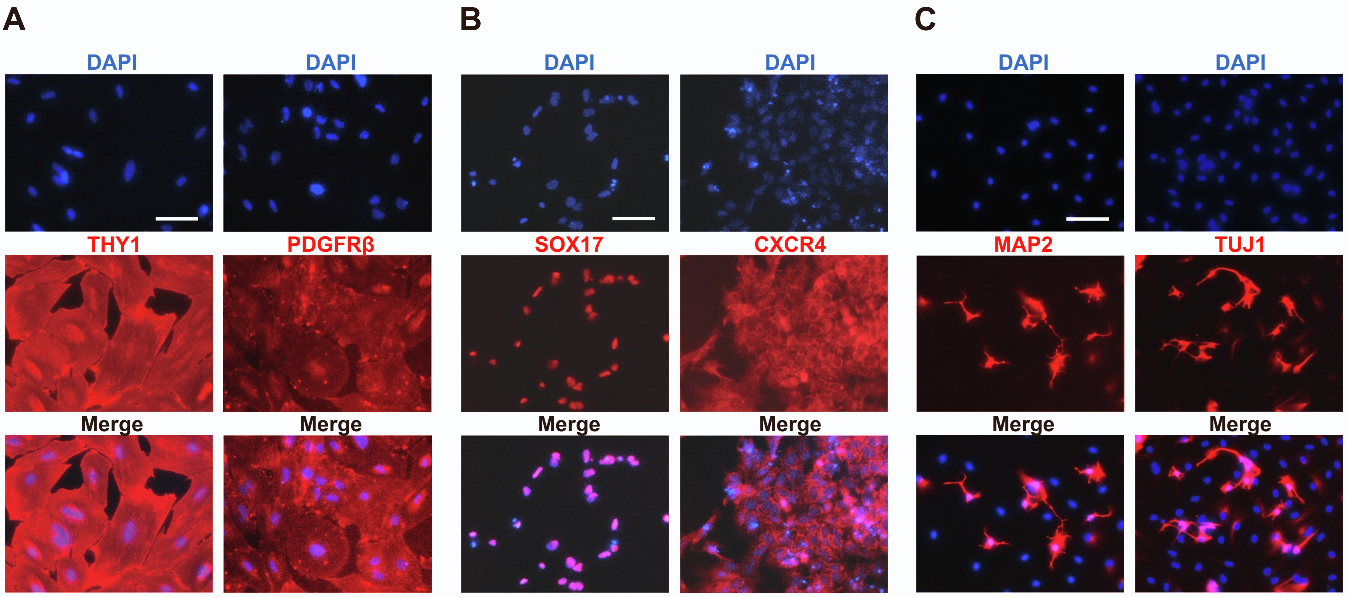

Figure S5. Differentiation of CA-p38-Induced Naïve hPSCs, Related to Figure 2.

(A) Representative Immunofluorescence staining for mesoderm markers THY1 and PDGFRβ.
(B) Representative Immunofluorescence staining for endoderm markers SOX17 and CXCR4.
(C) Representative Immunofluorescence staining for ectoderm markers MAP2 and TUJ1.
Scale bars: 100 μm.

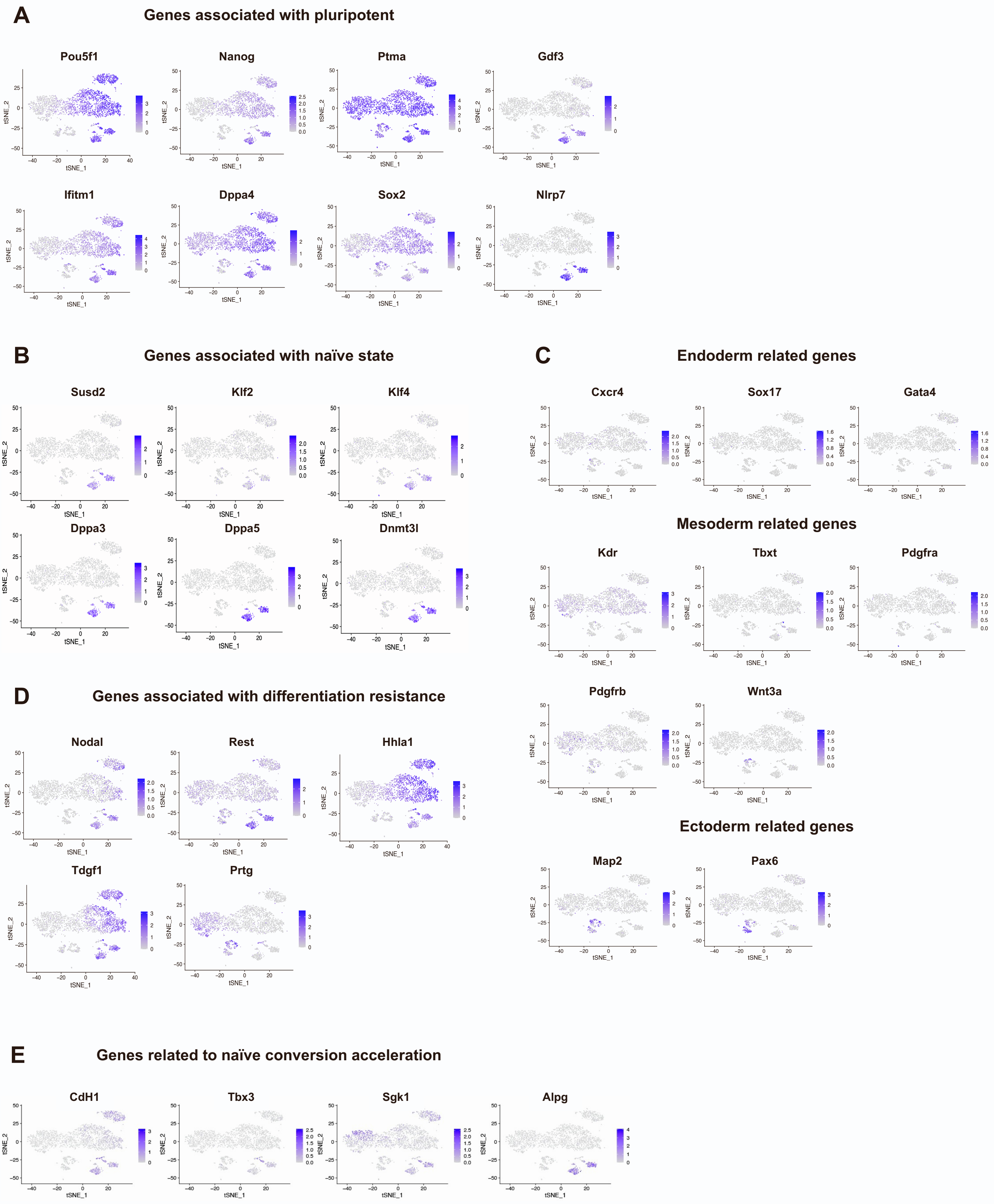
A
Genes associated with pluripotent
Pou5f1
Nanog
Ptma
Gdf3
Ifitm1
Dppa4
Sox2
Nlrp7
B
Genes associated with naïve state
Susd2
Klf2
Klf4
Dppa3
Dppa5
Dnmt3l
C
Endoderm related genes
Cxcr4
Sox17
Gata4
Mesoderm related genes
Kdr
Tbxt
Pdgfra
Pdgfrb
Wnt3a
Ectoderm related genes
Map2
Pax6
D
Genes associated with differentiation resistance
Nodal
Rest
Hhla1
Tdgf1
Prtg
E
Genes related to naïve conversion acceleration
CdH1
Tbx3
Sgk1
Alpg
tSNE_1
tSNE_2

Figure S6. Gene Expression Mapping in t-SNE Space During Naïve Conversion, Related to Figure 6.

(A) t-SNE mapping of pluripotency-related genes.
(B) t-SNE mapping of naïve-state-related genes.
(C) t-SNE mapping of differentiation-related genes.
(D) t-SNE mapping of differentiation-resistance-related genes.
(E) t-SNE mapping of genes associated with accelerated naïve conversion.

A

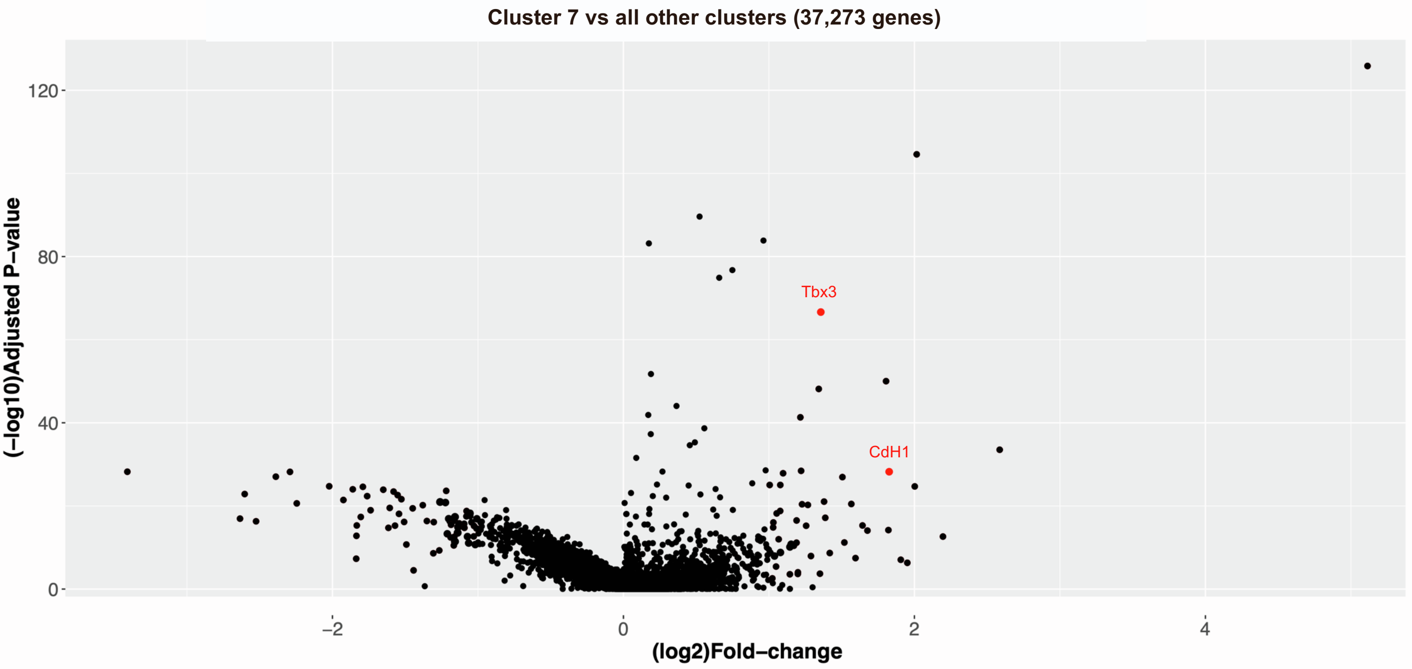

Cluster 7 vs all other clusters (37,273 genes)
(−log10)Adjusted P−value
120
80
40
0
−2
0
2
4
(log2)Fold−change
Tbx3
CdH1

B

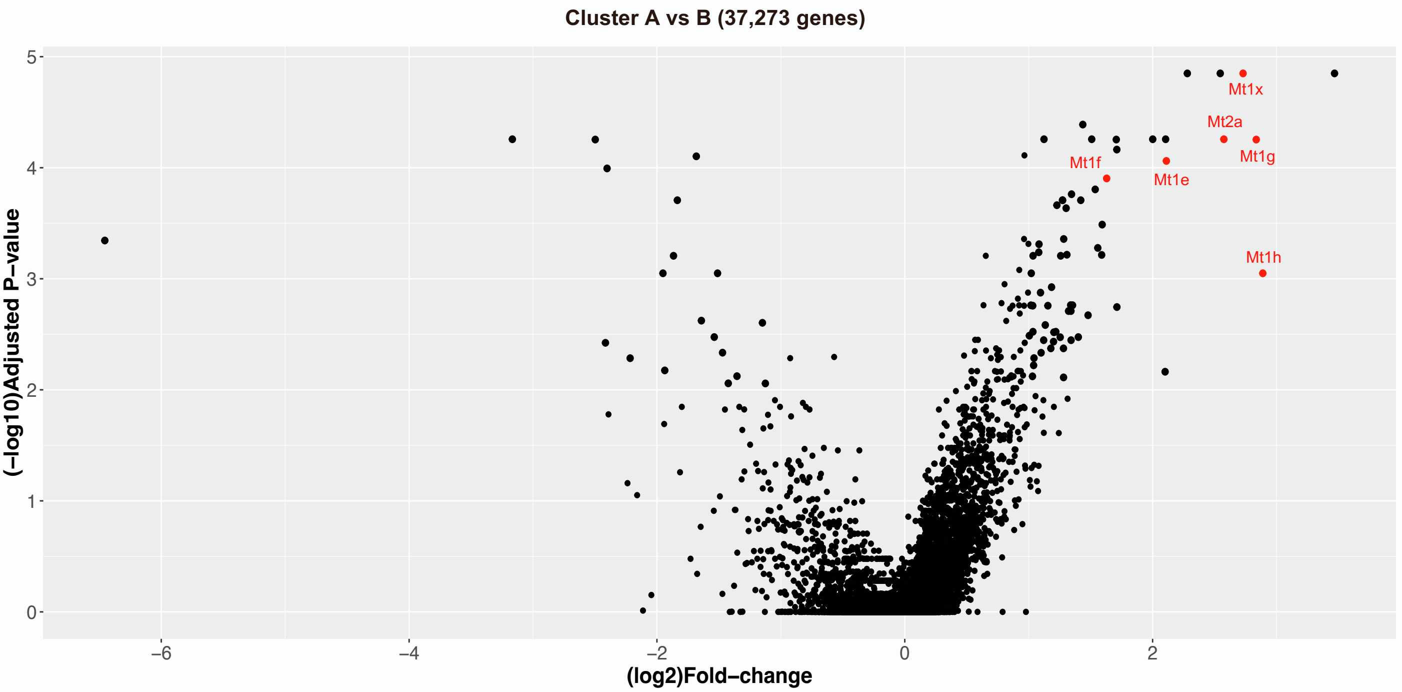

Cluster A vs B (37,273 genes)
(−log10)Adjusted P−value
5
4
3
2
1
0
−6
−4
−2
0
2
(log2)Fold−change
Mt1x
Mt2a
Mt1g
Mt1f
Mt1e
Mt1h

Figure S7. Differential Gene Expression Analysis During Naïve Conversion, Related to Figures 6 and 7.

(A) Volcano plot comparing cluster 7 with all other clusters (37,273 genes). The x-axis shows log2 fold change, and the y-axis shows the log10-adjusted p-value. Genes related to naïve conversion are highlighted in red.
(B) Volcano plot comparing gene expression between cluster A and cluster B (37,273 genes). The x-axis shows log2 fold change, and the y-axis shows the log10-adjusted p-value. Metallothionein-related genes are highlighted in red.

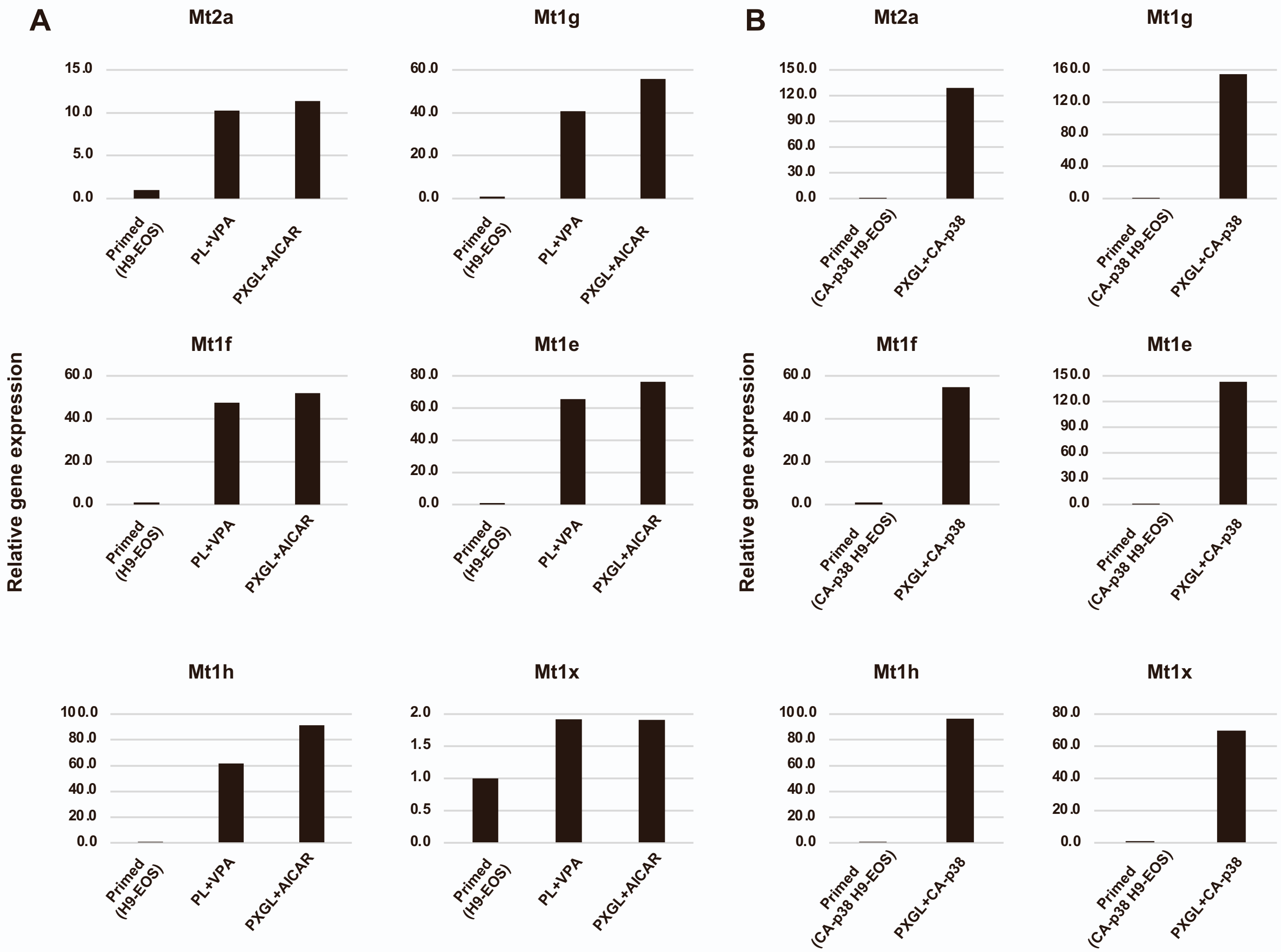

A
B
Mt2a
Mt1g
Mt1f
Mt1e
Mt1h
Mt1x
Relative gene expression
Primed (H9-EOS)
PL+VPA
PXGL+AICAR
Primed (CA-p38 H9-EOS)
PXGL+CA-p38

Figure S8. Metallothionein Gene Expression in Naïve hPSCs, Related to Figure 7.

(A) RNA expression analysis of sorted SUSD2$^+$CD75$^+$ cells versus parental primed hESCs (H9-EOS). VPA: day9+12p, AICAR: day14+15p.
(B) RNA expression analysis of sorted SUSD2$^+$CD75$^+$ cells versus parental primed hESCs (CA-p38 H9-EOS). CA-p38: day7+12p.

Table S1. Comparison of Naïve Conversion Protocols and Media Compositions, Related to Figure 1.

		Naïve hPSC						induced Naïve hPSC															
		2016	2019	2021				2013	2013	2014	2014	2016	2017	2019	2020	2021			2021			2022	
		Guo et al.	Bredenkamp et al.	Khan et al.				Gafni et al.	Chan et al.	Takashima et al.	Theunissen et al.	Qin et al.	Guo et al.	Szczerbinska et al.	Hu et al.	Bayerl et al.			Khan et al.			this study	
		Stem Cell Reports	Stem Cell Reports	Cell reports				nature	Cell stem cell	Cell	Cell stem cell	Cell reports	Development	Stem Cell Reports	SCIENCE ADVANCES	Cell stem cell			Cell reports				
		HNES (t2iLGoY)	HNES (PXGL)	FXGY	AXGY	PXGY	GXGY	NHSM	3iL	Reset cell	5i/L/A	Yin-PSCs	chemically reset (cR)	FINE cell	2iLI	HENSM	tHENSM	0HENSM	FXGY/A	AXGGY/A	PXGGY		
maintaining for naïve state related component	Medium	N2B27 medium	N2B27 medium	N2B27 medium				knockout DMEM,N2 supplement, etc. medium	TeSR1 medium	N2B27 medium	N2B27 medium	N2B27 medium	N2B27 medium	N2B27 medium	N2B27 medium	DMEM-F12, N2 supplement, etc. medium	DMEM-F12, N2 supplement, etc. medium	DMEM-F12, N2 supplement, etc. medium	N2B27 medium			N2B27 medium	
	Growth factors	LIF (10 ng/ml)	LIF (10 ng/ml)					LIF (20 ng/ml)	LIF (10 ng/ml)	LIF (10 ng/ml)	LIF (10 ng/ml)	LIF (10 ng/ml)	LIF (10 ng/ml)	LIF (20 ng/ml)	LIF (20 ng/ml)	LIF (20 ng/ml)	LIF (20 ng/ml)	LIF (20 ng/ml)				LIF (10 ng/ml)	
	FGFR inhibitor			PD166866 (1 µM)															PD166866 (1 µM)				
	Raf inhibitor				AZ628 (5 µM)														AZ628 (5 µM) *	AZ628 (5 µM)			AZ628 (5 µM)
	MEK inhibitor																						
	ERK inhibitor	PD0325901 (1 µM)	PD0325901 (1 µM)			PD0325901 (1 µM)	GDC-0994 (5 µM)	PD0325901 (1 µM)	PD0325901 (1 µM)	PD0325901 (1 µM)	PD0325901 (1 µM)	PD0325901 (0.5 µM)	PD0325901 (1 µM)	PD0325901 (1 µM)	PD0325901 (1 µM)	PD0325901 (1 µM)	PD0325901 (0.33 µM)		GDC-0994 (2.5 µM) *		PD0325901 (1 µM)	PD0325901 (1 µM)	
	GSK3 inhibitor	CHIR99021 (1 µM)	—					CHIR99021 (3 µM)	BIO (2 µM)	CHIR99021 (1 µM)	IM-12 (1 µM)	CHIR99021 (3 µM)	CHIR99021 (0 or 0.3 µM)	—	CHIR99021 (3 µM)							—	
	Tankyrase inhibitor	—	XAV939 (2 µM)	XAV939 (2 µM)	XAV939 (2 µM)	XAV939 (2 µM)	XAV939 (2 µM)	—	—	—	—	—	XAV939 (2 µM)	—	—	XAV939 (2 µM)	XAV939 (2 µM)	XAV939 (2 µM)	XAV939 (2 µM)	XAV939 (2 µM)	XAV939 (2 µM)	XAV939 (2 µM)	XAV939 (2 µM)
	ROCK inhibitor	Y-27632 (10 µM)	—	Y-27632 (10 µM)	Y-27632 (10 µM)	Y-27632 (10 µM)	Y-27632 (10 µM)	Y-27632 (5 µM)	—	—	Y-27632 (10 µM)	—	—	Y-27632 (10 µM)	—	Y-27632 (1.2 µM)			Y-27632 (10 µM)	Y-27632 (10 µM)	Y-27632 (10 µM)	—	Y-27632 (10 µM) *
	PKC inhibitor	Go6983 (2.5 µM)	Go6983 (2 µM)	Go6983 (5 µM)	Go6983 (5 µM)	Go6983 (5 µM)	Go6983 (5 µM)	Go6983 (5 µM)	—	Go6983 (5 µM)	—	—	Go6983 (5 µM)	—	—	Go6983 (2 µM)	Go6983 (2 µM)	Go6983 (2 µM)	Go6983 (5 µM)	Go6983 (5 µM)	Go6983 (5 µM)	Go6983 (2 µM)	Go6983 (5 µM)
induction for naïve state related component	Growth factors	—	—	—	—	—	—	FGF2 (8 ng/ml)	—	—	—	—	—	FGF2 (8 ng/ml)	—							—	—
	Cytokines	—	—	—	—	—	—	TGFβ1 (1 ng/ml)	—	—	Activin (20 ng/ml)	—	—	Activin (20 ng/ml)	—	Activin (5 - 20 ng/ml) *			Activin (10 ng/ml)	Activin (10 ng/ml)	Activin (10 ng/ml) *	—	—
	B-Raf kinase inhibitior	—	—	—	—	—	—	—	—	—	SB590885 (0.5 µM)	—	—	SB590885 (0.5 µM)	—							—	—
	Other inhibitors	—	—	—	—	—	—	JNK inhibitor (SP600125 10 µM)	—	–	Src inhibitor (WH4023 1 µM)	—	—	—	mTOR inhibitor (Torin1 10 µM)	Src inhibitor (CGP77675 1.2 µM)	Notch inhibitor (DBZ 0.15 µM)	Notch inhibitor (DBZ 0.15 - 0.3 µM)		ERK inhibitor GDC-0994 (2.5 µM)	ERK inhibitor GDC-0994 (2.5 µM)	—	—
	Transgene	—	—	—	—	—	—	—	—	Nanog<br>KLF2	—	(YAP ※)	—	—	—							(CA-p38 ※)	
	AMPK-p38	—	—	—	—	—	—	p38 inhibitor<br>(SB203580 10 µM)	AMPK inhibitor<br>(Dorsomorphin 2 µM)	—	—	cAMP, AMPK activator<br>(Forskolin 10 µM)	—	—	—	p38 inhibitor<br>(BIRB0796 0.8 µM)						AMPK activator<br>(AICAR 1mM)	AMPK activator<br>(AICAR 1mM or Metformin 5mM)
	Others	—	—	—	—	—	—	—	—	—	—	YAP activator<br>(LPA 10 µM)	Valproic acid sodium salt (1mM)	AZD5438 (0.1 µM)<br>Dasatinib (0.1 µM)	human insulin<br>(18 µg/ml)							–	–
DNA CpG methylation		Low	—	—	Low	—	—	Low	—	Low	—	—	Low	Low	Low	Low	High	High	—	—	Low	Low	—
Repressive histone marks		—	—	—	—	—	—	H3K27me3: low	H3K27me3: low	H3K9me3: low<br>H3K27me3: low	H3K27me3: low	H3K9me3: low	—	H3K9me3: low	H3K27me3: low	—	—	—	—	—	—	H3K9me3: low	—
Predominant OCT4 enhancer		—	—	Distal	Distal	Distal	Distal	Distal	—	Distal	Distal	—	Distal	—	Distal	Distal	Distal	Distal	Distal	Distal	Distal	Distal	Distal
cell surcafece marker		—	CD7, CD75, CD77, CD130, SUSD2	CD75, SUSD2	CD75, SUSD2	CD75, SUSD2	CD75, SUSD2	E-CAD	—	—	—	—	—	CD75, CD130	—	SUSD2, CD24, CD130, CD77	—	—	CD75, SUSD2	CD75, SUSD2	CD75, SUSD2	CD75, SUSD2	CD75, SUSD2
transcriptome		KLF4, TFCP2L1, KLF17, STELLA, DPPA5, KLF2, TBX3	KLF17, TFCP2L1, KLF4, DPPA5	DPPA3, KHDC3L, DNMT3L, NLRP2, DPPA5, KLF17, TFCP2L1, KLF5, KLF4	DPPA3, KHDC3L, DNMT3L, NLRP2, DPPA5, KLF17, TFCP2L1, KLF5, KLF4	DPPA3, KHDC3L, DNMT3L, NLRP2, DPPA5, KLF17, TFCP2L1, KLF5, KLF4	DPPA3, KHDC3L, DNMT3L, NLRP2, DPPA5, KLF17, TFCP2L1, KLF5, KLF4	—	GP130, KLF4, TBX3, STELLA, KLF5	TBX3, REX1, STELLA, TFCP2L1, KLF2, KLF4, GBX2, ESRRB	KLF2, KLF4, REX1, STELLA, DPPA5, TFCP2L1	GP130, TBX3, TFCP2L1, STELLA, HERVH-Gap, HERVH-Pol	KLF17, TFCP2L1, STELLA, KLF4, TBX3	KLF4, KLF5, KLF17, STELLA, LTR7Y, TBX3	KLF4, KLF2	KLF4, KLF17, DPPA5, STELLA, DNMT3L, REX1, XIST, TFCP2L1, KHDC1L	KLF4, KLF17, DPPA5, DNMT3L, REX1, TFCP2L1, KHDC1L	KLF4, KLF17, DPPA5, STELLA, DNMT3L, REX1, TFCP2L1, KHDC1L, FGF4	—	—	DPPA3, KHDC1L, NLRP2, DNMT3L, DPPA5, KLF17, TFCP2L1, KLF5, KLF4	KLF2, KLF4, KLF17, TFCP2L1, STELLA	—
TFE3 nuclear localization		—	—	—	—	—	—	YES	—	YES	—	—	—	YES	YES	YES	—	—	—	—	—	YES	—
Mitochondrial membrane activity and depolarization		High	—	—	—	—	—	—	—	High	—	—	—	—	High	High	—	—	—	—	—	High	—

* Optional factors

Table S2. Primer Sequences for qPCR, Related to STAR Methods, Figure 1 and S3

Gene	Forward primer 5’-3’	Reverse primer 5’-3’
RPS18	ACTCAACACGGGAAACCTCA	AACCAGACAAATCGCTCCAC
OCT3/4	TGTACTCCTCGGTCCCTTTC	TCCAGGTTTTCTTTCCCTAGC
NANOG	CAGTCTGGACACTGGCTGAA	CTCGCTGATTAGGCTCCAAC
KLF4	GATGGGGTCTGTGACTGGAT	CCCCCAACTCACGGATATAA
TFCP2L1	CTCAGGTGCTGACTTGCTGA	ATGGCGTGGTACACAGACAG
STELLA	TCTCCACAAATGCTCACCGA	TCTTCTTTCATGCGTACGAACTCC
KLF2	CATCTGAAGGCGCATCTG	CGTGTGCTTTCGGTAGTGG
